# IMU-based joint axis identification method for arbitrary joints in OpenSim - a simulation study

**DOI:** 10.1186/s42490-025-00102-7

**Published:** 2025-11-21

**Authors:** Iris Wechsler, Julian Shanbhag, Sandro Wartzack, Anne D. Koelewijn, Jörg Miehling

**Affiliations:** 1 Engineering Design, Friedrich-Alexander-Universität Erlangen-Nürnberg, Martensstrasse 9, 91058 Erlangen, Germany; 2 Machine Learning and Data Analytics Lab, Friedrich-Alexander-Universität Erlangen-Nürnberg, Carl-Thiersch-Strasse 2b, 91052 Erlangen, Germany; 3 Chair of Autonomous Systems and Mechatronics, Friedrich-Alexander-Universität Erlangen-Nürnberg, Paul-Gordan-Strasse 3/5, 91052 Erlangen, Germany

**Keywords:** Biomechanical modeling and simulation, Joint axis identification, Model individualization, Instantaneous axis of rotation, Measurement noise

## Abstract

In musculoskeletal simulation, individualized joint axes enhance the accuracy and reliability of kinematic and kinetic simulation results. We investigated the correctness and performance of an analytical method for identifying the instantaneous axis of rotation between two bodies based on motion data in OpenSim. The instantaneous center of rotation is the point at which two bodies have the same velocity. The relative linear and angular velocity between the two bodies, as well as their relative position to each another, are required as inputs to calculate it. Using the instantaneous center of rotation, fixed or moving joint centers of rotation can be identified. To prove the general applicability of the method, the instantaneous centers of rotation of a revolute joint of a simple double pendulum model and the hip and knee joint of a more complex musculoskeletal model were investigated. The hip joint is defined as a ball joint. The knee joint is defined as an OpenSim custom joint which describes the motion of the child segment in relation to the parent segment as a function of generalized coordinates. To verify the correctness of the approach in OpenSim, the moving centers of rotation were calculated using synthetic noisefree data. The results were compared to the implementation of the respective joints in the model which act as the ground truth. White Gaussian noise was added to the synthetic data to analyze its effect on the quality of the calculated centers of rotation. We were able to correctly identify the center of rotation of each joint using noisefree data. In the case of noisy data, joint centers of rotation can be determined by applying additional filtering or optimization methods to the calculated instantaneous centers of rotation. Consequently, we are able to determine the center of rotation for arbitrary joints based on noisy synthetic data. This approach is applicable for both fixed and moving centers of rotation which distinguishes it from commonly used methods in the field of biomechanical simulation.

**Supplementary Information:**

The online version contains supplementary material available at 10.1186/s42490-025-00102-7.

## Introduction

Human anthropometry is highly variable. This can be observed visually in measures such as a person’s weight, height or limb length. However, there are also differences in aspects that are not easily observable, such as muscle parameters, a person’s center of mass or joint axes. Individual movement patterns and the forces in the body that drive them are also not easily observable. These forces and other biomechanical parameters and variables can be determined using musculoskeletal simulations. Musculoskeletal models are needed which describe the human body as a multi-body system. The simulations can be used to answer (research) questions in a variety of fields. In a clinical context, musculoskeletal simulations are used to support the rehabilitation of patients [1–3], surgical planning [4, 5] or implant design [6–8]. In the field of sport science, they are used to analyze and optimize the performance of athletes [9, 10], and in the field of ergonomics, they are used to improve the ergonomic design of products [11, 12]. For all fields, the simulations should depict reality as closely as possible, otherwise the results may not be reliable and should not be used to answer the (research) questions posed.

To compute musculoskeletal variables, the inverse approach can be used. This approach requires experimentally measured motion data as input. Marker-based optical motion capture systems depict the gold standard for collecting motion data. However, alternative systems such as inertial measurement units (IMUs) or depth-cameras can also be used. Inverse kinematics is used to transfer the experimentally measured motion data onto the musculoskeletal models. By convention, optimizations are used to compute the generalized coordinates – mostly joint angles – which describe the motion of the model. Both marker-based and IMU-based experimental measurements are affected by measurement noise. Marker position measurements are influenced by random noise and systematic errors, such as soft-tissue artefacts. IMUs are prone to sensor drift. Regardless of the measurement system used, optimizations calculate joint angles by identifying the optimal match between the experimental sensor data (e.g. marker positions or IMU orientations) and the virtual sensors placed on the model [13–16]. The greater the measurement error is, the greater the deviation between the actual movement – performed by the participant – and inverse kinematics result will be. In addition to the experimental measurement data, the musculoskeletal models used also influence the accuracy and reliability of simulation results.

Individualized musculoskeletal models enhance the accuracy of computed kinematic and kinetic parameters in biomechanical simulations [17]. Various model parameters can be individualized to achieve more accurate simulation results (e.g. segment lengths and body segment inertial parameters). The standard approach is to scale a generic musculoskeletal model so that the segment lengths of the model match the anthropometry of the person whose motion was measured experimentally. Scaling segment lengths also scales inertial parameters, such as segment mass, center of mass and moments of inertia, of all segments accordingly. A target mass can be entered to enhance the segment mass scaling results. To adjust the mass of the model, each segment mass is scaled using the scaling factor calculated on the basis of the marker data. Should the total mass of the model exceed the target value, each segment mass is scaled again in accordance with the ratio of the target mass to the summed scaled masses. In OpenSim – a framework for musculoskeletal simulations [18] – the joint axes and centers of rotation are predefined in the model. No further individualization occurs, except that the scaling factor of the corresponding segment the joint is defined in is also applied to the joint frame positions. This may lead to poor tracking results which may then result in inaccurate kinematic simulation results. Additionally, computed kinetic results, such as joint torques, may be negatively affected.

Camomilla et al. [19] investigated factors of biomechanical simulations that affect esimated joint torques in clinical gait analysis within the framework of a systematic literature review. The authors reported that joint axes and joint center positions affect muscle moment arms and joint torques as well as their interpretation. For the hip joint, a $30 \, mm$ hip joint center calculation error can lead to a mean error of $22 \%$ and $15 \%$ for the flexion-extension and abduction-adduction joint torques, respectively [20–23]. For the knee joint, an error of $\pm 10 \, mm$ in the knee joint center can lead to a variation of the knee flexion-extension moment from $0.7 \, bodyweight \, (BW) \cdot height \, (H)$ [24] to up to $0.92 \, BW \cdot H$ [22]. Most et al. [25] reported that knee angle kinematics were sensitive to the implemented flexion-extension axis of the model and Reinbolt et al. [22] concluded that variations in axis positions and orientations in body segments significantly affected lower-body joint torques with a maximum root mean square error (RMSE) of $4 \%\:BW \cdot H$. Consequently, for musculoskeletal simulations, the individualization of joint rotation axes and centers is important to achieve accurate and reliable kinematic and kinetic results. 

In the field of musculoskeletal simulation, various methods have been used to identify individualized joint axes and centers of rotation to enhance the accuracy of musculoskeletal simulation results and specifically calculated joint torques. Both marker-based and IMU-based approaches exist. Marker-based approaches use optimization methods to identify joint centers and axes of rotation based on experimental measurements. Most methods assume the center of rotation to be stationary and therefore require one segment to be stationary [26–29]. For other methods, both segments may be in motion [30]. Ehrig et al. [30] investigated the performance of different methods for identifying the center of rotation of a virtual hip joint using synthetic motion data. To simulate experimental marker measurements, white Gaussian noise as well as a systematic noise – representing soft tissue artifacts – has been added to the virtual data. For all techniques, the RMSE between the known and estimated joint center increased with a decreasing range of motion and most techniques limited the center of rotation to an RMSE within $0.3\,cm$ when the range of motion was greater than $45^{\circ}$.        

As IMUs gain increasing popularity in musculoskeletal simulations because of their low costs, flexible application and ease of use [31–35], IMU-based joint identification approaches have been proposed. The approaches use either optimization methods or a Kalman filter to estimate stationary centers of rotation. Using the Kalman Filter [36], the center of rotation for synthetic motion data with added white Gaussian noise was identified with a mean error smaller than $3.5 \%$. Using optimization methods [37], the center of rotation of a double pendulum was identified based on experimentally measured IMU-based motion data. An Euclidean error between $0.07 - 0.23\:mm$ was reported.

All these approaches are limited because they assume the center of rotation to be fixed in relation to a body coordinate system. However, there are joints in the human body where this assumption does not hold true. For example, the center of rotation of the knee joint varies depending on the knee angle. We refer to this as a moving center of rotation. This can be observed by the motion of the tibia in relation to the femur bone during knee flexion motion [38]. Using available approaches, individualized axes of rotation for the knee joint cannot be estimated based on experimentally measured motion data.

All previously described methods have in common that the center or axis of rotation is identified either by means of optimization problems or by using an extended Kalman Filter. However, in the wider field of engineering, there are also methods available to calculate the instantaneous center of rotation (ICOR) of a moving rigid body, either indirectly or directly. The motion of a rigid body in a plane consists of translation and rotation. However, at any time, it can also be interpreted as a pure rotational movement around a momentary center of rotation, the ICOR [39]. One major advantage is that the ICOR can be determined for two moving body segments and consequently for a moving center / axis of rotation. Kunz [40] derived a direct, analytical method for calculating the ICOR for a three-dimensional rigid body relying on the linear and angular velocity of the body. The author provides a detailed description how to calculate the ICOR. It was not employed with a specific application in mind. The method is presented for both two-dimensional and three-dimensional applications. As the method relies on linear and angular velocity data of body segments, which are readily available parameters in musculoskeletal simulation, using this approach for identifying both fixed and moving joint axes and centers of rotations for musculoskeletal simulations seems logical. However, the feasibility and performance of calculating the ICOR for identifying arbitrary joint axes and centers of rotation in musculoskeletal models has not yet been analyzed.

In this contribution we investigate the feasibility of the ICOR calculation method presented by Kunz [40] to identify the moving center of rotation from motion data for arbitrary joints in OpenSim. To verify the correctness of the approach, the moving axes/centers of rotation were calculated using noisefree synthetic data and compared to the implementation of the respective joints in the model. We thereafter added white Gaussian noise to the virtual sensor data to analyze the effect of noisy measurement data on the quality of calculated moving axes/ centers of rotation. To prove the general validity and applicability to identify moving axes/center of rotations from noisy data, two different models, a simple double pendulum with a revolute joint and two joints in a more complex full-body musculoskeletal models were analyzed. For the musculoskeletal model, a ball joint and a custom knee joint were analyzed.

## Methods

### IAOR, ICOR and their relation to human joints

As the principle of an ICOR and instantaneous axis of rotation (IAOR) is essential for the understanding of this article, we give a brief overview of the difference between the ICOR/IAOR and a moving center or axis of rotation as well as its relation to the modeling of joints in virtual models.

In two-dimensional motion, the ICOR can be used to interpret the motion of a rigid body as a pure rotation. The same motion can be described by a moving center of rotation and the rotation around this center. For fixed centers of rotation, the ICOR at any instant is the same as the center of rotation. However, for moving centers of rotation, this does not apply. The sum of all instantaneous centers of rotation then describes the course of the moving center of rotation. For three-dimensional motions, the ICOR expands to an IAOR as a rigid body may translate and rotate in three dimensions, respectively.

In the human body, there are joints with a fixed center of rotation (e.g. the hip joint), but there are also joints with a variable axis of rotation (e.g. the knee joint). The modeling of these joints in the musculoskeletal model is described in Section “Process description”. Consequently, the principle of the ICOR and IAOR can be used to identify and define the modeled joint axes and centers.

### IAOR calcuation method

Kunz [40] describes a direct, analytical method to calculate the IAOR of a moving rigid body relative to a fixed coordinate system. For planar movements the ICOR ($\vec{r}_{C/O}$) can be calculated using equation 1:

1 $$\vec{r}_{C/O} = \frac{\vec{\omega}_{B/F} \times \vec{\nu}_{p/O}}{{\lVert \vec{\omega}_{B/F} \rVert}^{2}} + \vec{r}_{p/O}$$

where $\vec{r}_{p/O}$ is a point on the body, $\vec{\omega_{B/F}}$ is the angular velocity of the body and $\vec{\nu_{p/O}}$ is the linear velocity of $\vec{r}_{p/O}$ on the body expressed in the fixed coordinate system $F$. For planar movements, $\vec{r}_{C/O}$ represents a unique point that is not affected by the choice of $\vec{r}_{p/O}$. However, when using this approach to calculate the ICOR for three-dimensional movements the point $\vec{r}_{C/O}$ is then dependent on the choice of the point $\vec{r}_{p/O}$, because the ICOR expands to an IAOR in three-dimensional space.

To calculate the ICOR ($\vec{r}_{C/B_{1}}$) between two moving rigid bodies, the following information has to be known: The relative linear ($\vec{\nu}_{rel}$) and angular ($\vec{\omega}_{rel}$) velocity between body 1 and body 2 and the position ($\vec{r}_{B_{2}/B_{1}}$) of body 2 in relation to body 1. The calculation works analogously to Eq. 1, only the fixed reference system is replaced by a moving body:

2 $$\vec{r}_{C/B_{1}} = \frac{\vec{\omega}_{rel} \times \vec{\nu}_{rel}}{{\lVert \vec{\omega}_{rel} \rVert}^{2}} + \vec{r}_{B_{2}/B_{1}}$$

For three-dimensional motions, the ICOR expands to an IAOR. The point $\vec{r}_{C/B_{1}}$ is then a point on the IAOR. The direction $\vec{e}$ of the IAOR can be calculated:

3 $$ \vec{e} = \frac{\vec{\omega}_{rel}}{ \lVert \vec{\omega}_{rel} \rVert}$$

and the IAOR can be described:

4 $$ IAOR = \vec{r}_{C/B_{1}} + t \vec{e} \qquad (t \in R)$$

Two models were used to examine the correctness and performance of the method for application in musculoskeletal modeling in OpenSim: a simple double pendulum model and a more complex musculoskeletal model. For both models, virtual (IMU) data was created using OpenSim.

### Double pendulum model

#### Model description

**Fig. 1:**
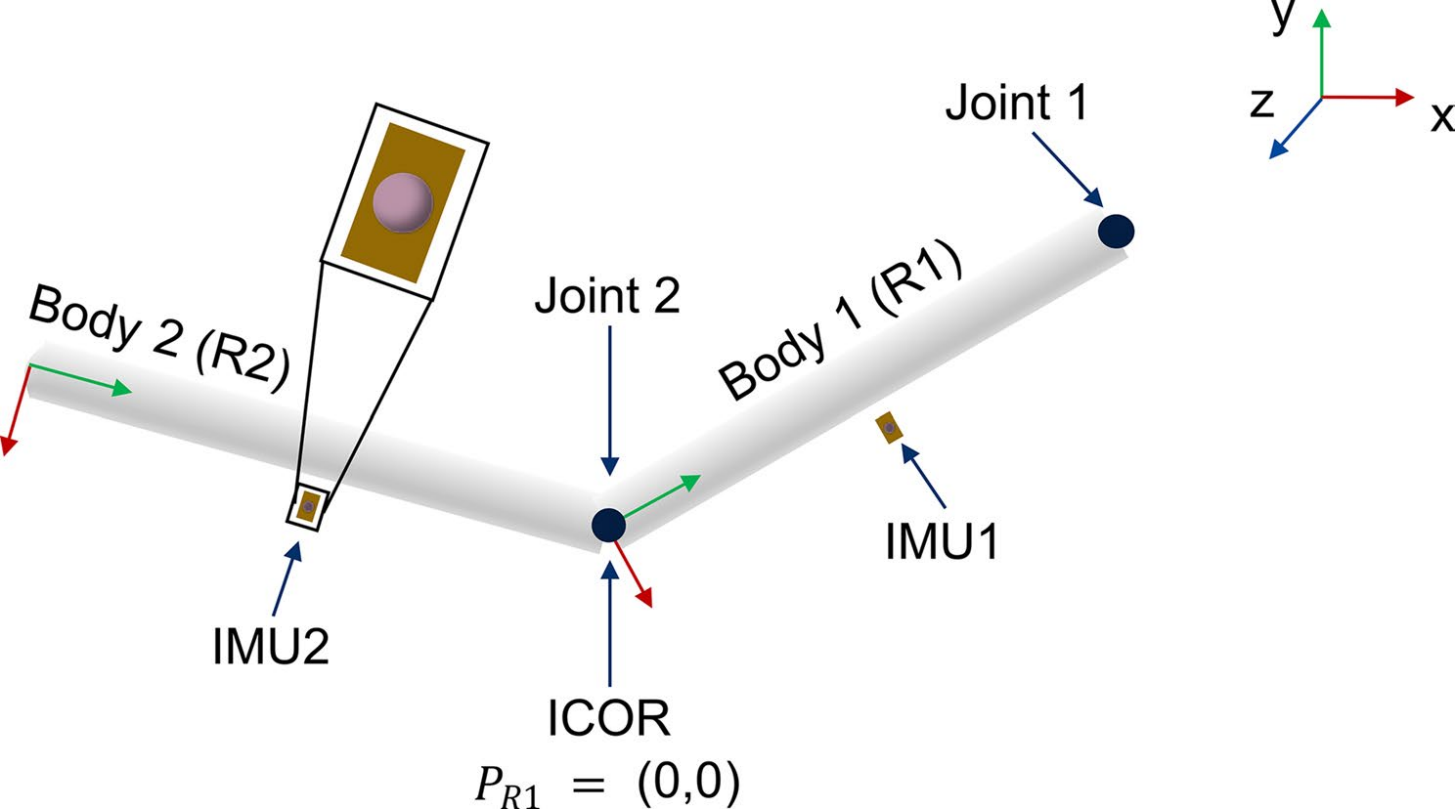
Two degree of freedom double pendulum model issued by OpenSim. Virtual IMUs and model markers are shown as orange boxes and pink circles respectively

To evaluate the theoretical correctness of the method, the center of rotation of a double pendulum model was identified using the ICOR. The planar double pendulum model and its components are shown in Fig. 1. The model consists of two bodies which are conjoined by a revolute joint that is placed in the origin of body 1 ($P_{R1} = (0,0)$). Additionally, body 1 is fixed in space (in the ground frame) using a revolute joint. On each body, one virtual IMU was attached at coordinate $(0.1,0.5,0)$. Model markers have been placed in the origin of each IMU coordinate system.

#### Process description

**Fig. 2:**
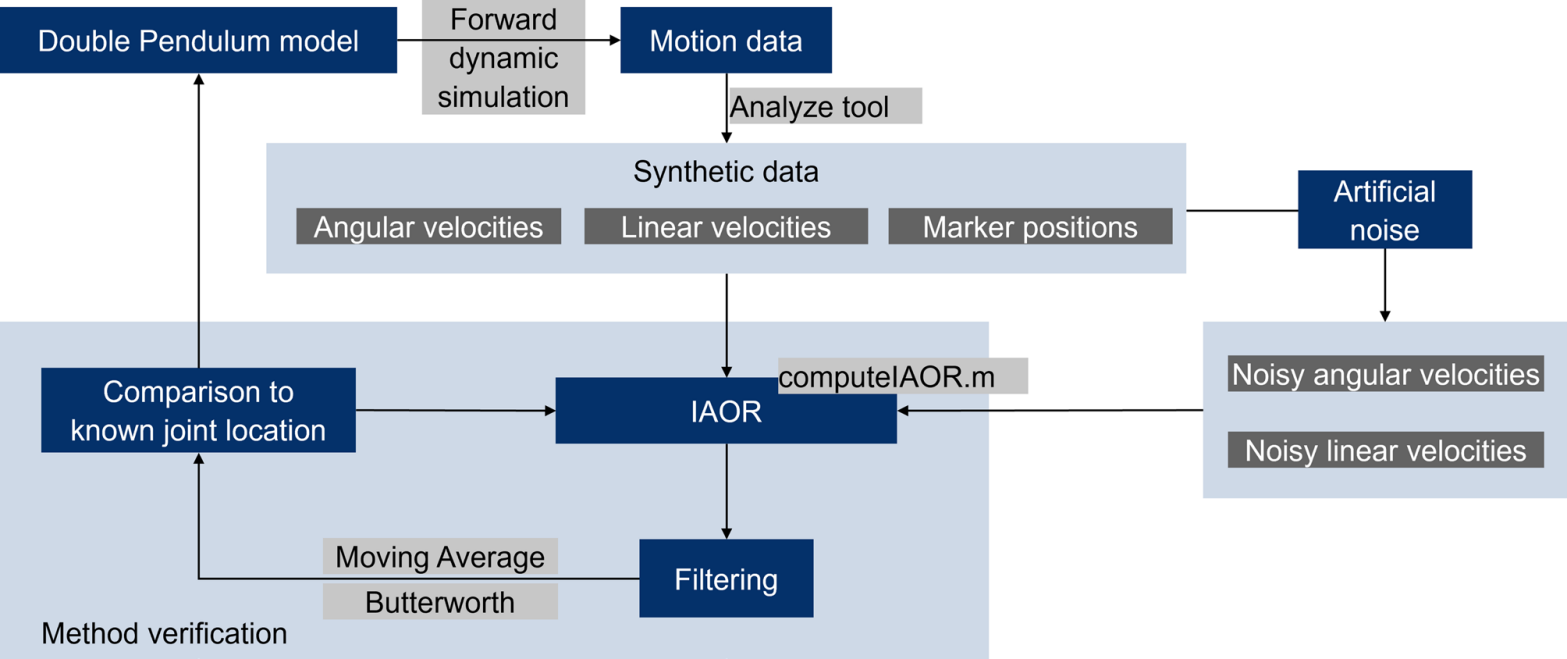
Workflow used to analyze the correctness and performance of the joint axis identification method using the example of the double pendulum model issued by OpenSim

**Fig. 3:**
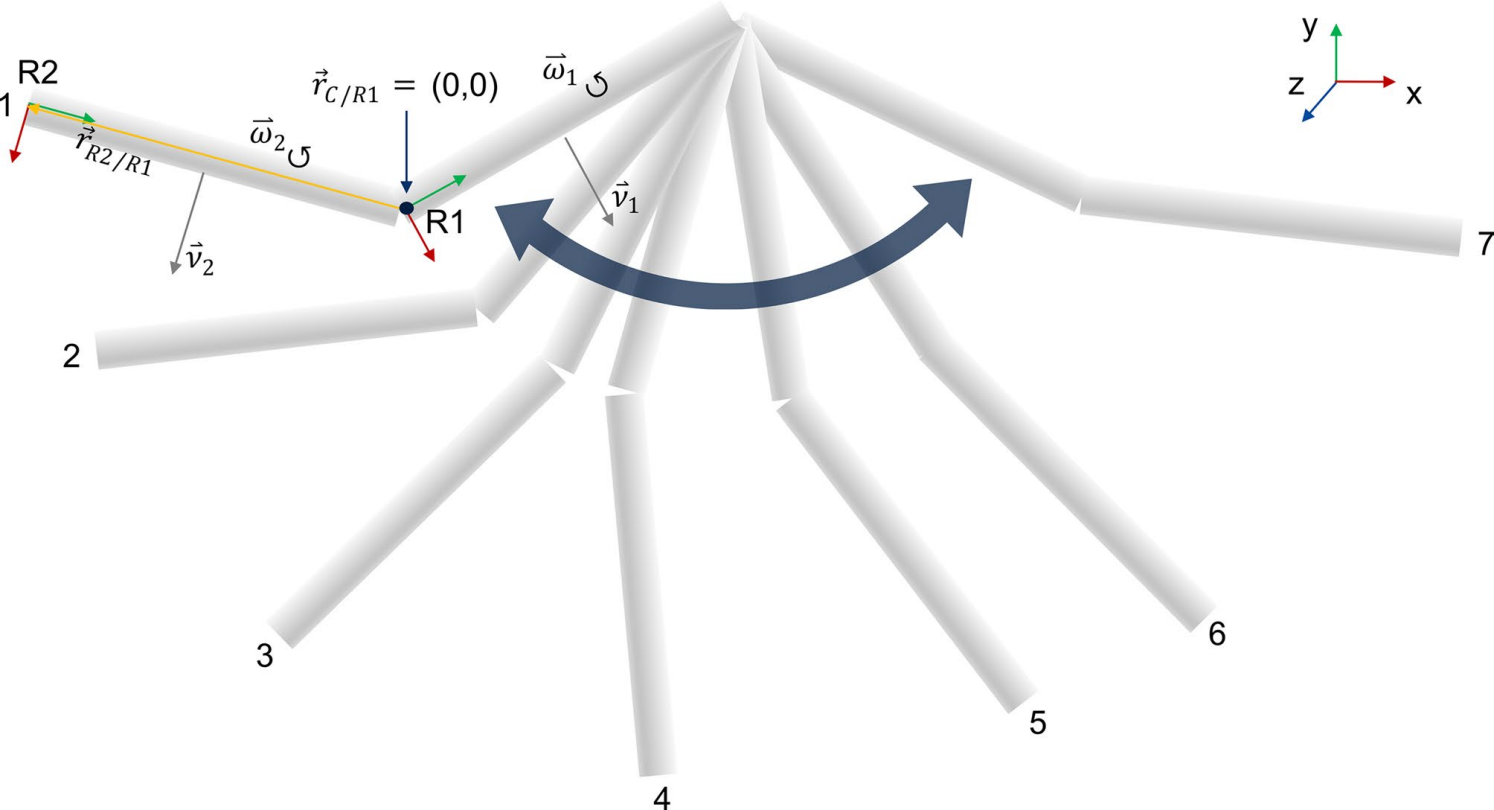
Simulated pendulum motion created using forward dynamic simulation and orientation and direction of variables needed as input for ICOR calculation. The numbers indicate the initial direction of the motion

Figure 2 provides an overview of the process used to analyze the correctness of the method using the double pendulum model as an example. The starting point of the process is the double pendulum model and associated Matlab script issued by OpenSim. OpenSim 4.4 was used to run a forward dynamic simulation. The sampling frequency was set at 100 Hz. The starting position of the model is shown in Figure 1. No other external forces besides gravity are defined to act on the model. The forward simulation created a motion showing the pendulum swinging back and forth. The motion is illustrated in Fig. 3. The pendulum swings back and forth a total of 3.5 times. Using this motion, the synthetic data, needed as input for the ICOR computation method, was created. The linear velocities of the IMUs and the position of the IMU markers as well as the IMU angular velocities and linear accelerations were calculated based on the generated motion data. The ICOR was computed using the synthetic data and the results were compared to the location of the joint between the two bodies which is known through the model and thus acts as the ground truth, see Fig. 1. Figure 3 illustrates and identifies variables that are needed to calculate the ICOR. $R1$ and $R2$ describe both bodies, $\vec{r}_{C/R1}$ is the ICOR expressed in the coordinate system of body 1, $\vec{\nu}$ and $\vec{\omega}$ describe the linear and angular velocity of both bodies respectively and $\vec{r}_{R2/R1}$ is the position of body 2 in the coordinate system of body 1. We visualized only the linear and angular velocity of both bodies and not the relative velocities between the bodies that are needed as input for Eq. 2, as these variables cannot be visualized. The ICOR is then calulated using Eq. 2:

$$\vec{r}_{C/R1} = \frac{\vec{\omega}_{rel} \times \vec{\nu}_{rel}}{{\lVert \vec{\omega}_{rel} \rVert}^{2}} + \vec{r}_{R2/R1}$$

To evaluate the effect of noisy input data on the accuracy of the calculated center of rotation artificial white Gaussian noise ($X \sim N (\mu, \sigma^{2})$) was added to the linear ($X \sim N (0, 0.1)$) and angular ($X \sim N (0, 0.01)$) velocity values of the virtual IMUs. The ICOR was calculated using the noisy velocity data. Outliers were removed from the data using the 1.5-fold of the interquartil distance. The coordinates of the ICOR as a function of time were then filtered using two different filtering methods: (1) a moving average filter (MA) with a window length of 10 and (2) a second order Butterworth filter (BW) with a cutoff frequency of $5 \, Hz$. The mean and median of the resulting ICOR coordinates were compared with the ground truth to quantify the effectiveness of the filtering methods. The RMSE between the known and calculated center of rotation for each coordinate was computed for both filtering methods.

### Musculoskeletal model

#### Model description

**Fig. 4:**
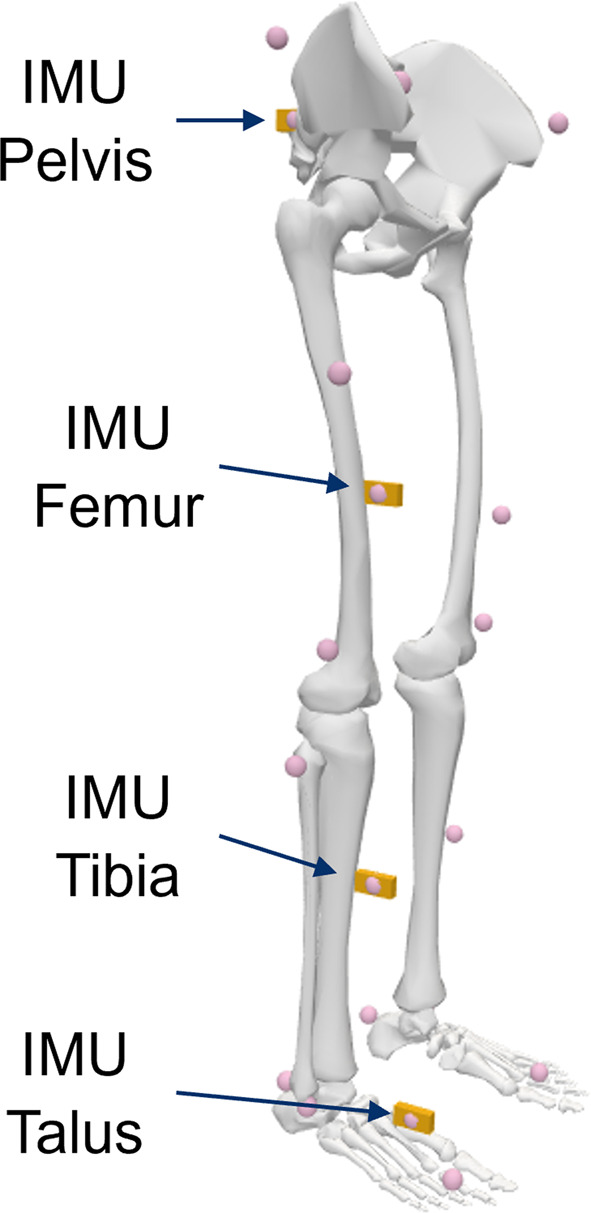
Lower body of the generic musculoskeletal model. Virtual IMUs and model markers are shown as orange boxes and pink circles respectively

**Fig. 5:**
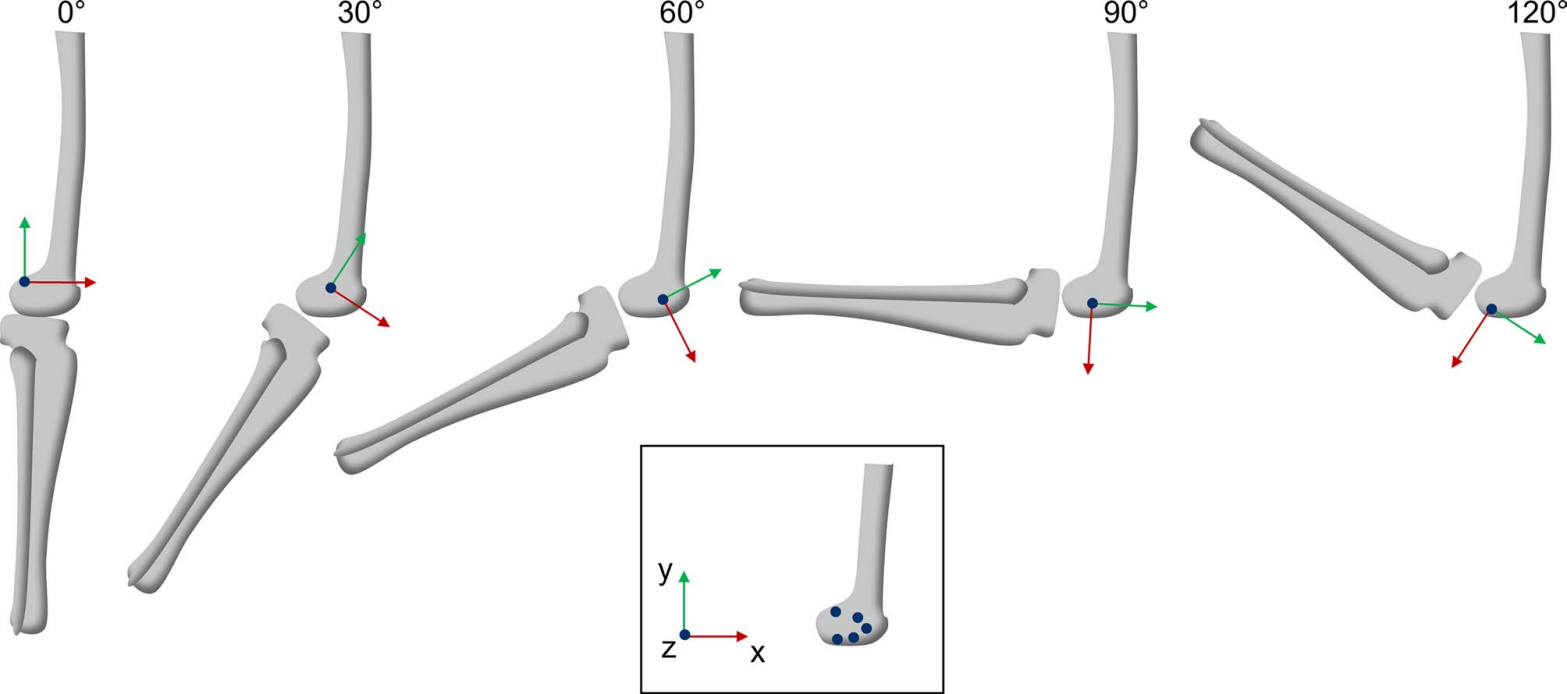
Position of knee joint axis (blue dot) for various knee angles. Framed in black, the position of the axis for each angle is shown in the femur

We identified the hip and knee joint center of rotation of a musculoskeletal model using the ICOR to prove the applicability of the method for identifying fixed and moving joint centers and axes of rotations in musculoskeletal models based on motion data. We used a generic full-body model for the investigation [41]. Virtual IMUs have been attached to the pelvis and the right upper and lower leg as well as the foot, see Fig. 4. For the musculoskeletal model two different joints are analyzed using the approach described in Section “IAOR calcuation method”: the knee joint and the hip joint. The hip joint is defined as a custom joint with three rotational degrees of freedom (ball joint). The knee joint is defined as a custom joint with one rotational and two translational degrees of freedoms. The translational degrees of freedom describe the translation of the tibia frame in relation to the femur frame as a function of the knee angle in the sagittal plane, see Fig. 5. Spline functions define these translations for custom joints in OpenSim.

#### Process description

The process for evaluating the joint axis identification method for the musculoskeletal model is in most steps analogous to the workflow for the double pendulum model described in Section “Process description”. Differences arise in the creation of virtual (IMU) data. Instead of computing a forward dynamic simulation, a motion file depicting a squat motion was used. The squat motion was selected as it encompasses a high range of motion in the knee joint. The sampling frequency of the motion file was 100 Hz. Experimental data acquisition and processing steps taken to create the squat motion file are described in Scherb et al. [42]. Again, the linear velocities of the IMUs and the position of the IMU markers as well as the IMU angular velocities and linear accelerations were calculated based on the generated motion data.

The fixed and moving centers of rotation of the hip and knee joint were computed using the virtual data. The result for the hip joint was compared to the implementation of the joint in the model. For the knee joint, the position of the tibia in the femur frame was calcualted based on the identified ICOR. The resulting motion curve of the tibia was then compared to the implemented splines in OpenSim. Artificial white Gaussian noise was added to the linear ($X \sim N (0, 0.02)$) and angular velocity ($X \sim N (0, 0.02)$) data of the virtual IMUs. The hip and knee joint differ in their implementation so different strategies are applied to identify the true ICOR from noisy data:

##### Hip joint

The ICOR is the point of intersection of the IAOR for every time step. This corresponds to the definition of the hip joint in the musculoskeletal model, as it is modeled as a three degree of freedom ball joint. To deal with the noisy data, the mean point of intersection is computed using a least squares approach and the resulting coordinate is compared to the known position of the hip joint center to quantify the effectiveness of the approach. The least squares approach calculates a point with minimum distance to each calculated axis of rotation by minimizing the sum of squared perpendicular distance between a point and all axes of rotation.

##### Knee joint

Custom joints in OpenSim are defined by describing the motion of the child segment in relation to the parent segment as a function of generalized coordinates. Thus, for the knee joint, not the ICOR itself but the coordinates (x,y) of the tibia frame in the femur frame as a function of the knee angle ($\phi$) are of interest. To deal with the noisy data, the coordinates are expressed as a function of time. Smoothing splines are applied to the noisy data. A margin of values surrounding the splines is defined and values of the ICOR coordinates outside this range are defined as outliers and removed. Afterwards, the ICOR coordinates are filtered using either a moving average filter with a window length of 10 or a third order Butterworth filter with a cutoff frequency of $5 \, Hz$. Again smoothing splines are applied to the filtered ICOR coordinates to identify the true course of the relation between each coordinate and the joint angle $\phi$. To quantify the effectiveness of the smoothing approach, the Pearson correlation coefficient between the noisefree and filtered curve is calculated for each coordinate. Then, based on the identified ICOR, the position of the tibia in relation to the femur frame is calculated beginning with a starting position.

The position of the tibia in the femur frame for a knee angle of zero is known from the scaled musculoskeletal model. This point is the starting position of the calculation. For specific knee angles, the ICOR of the knee joint is known. To calculate the new position of the tibia in the femur frame, the tibia frame is rotated by a specific angle around the identified center of rotation:

5 $$ \vec{b}_{n} = R(\vec{b}_{n-1} - \vec{c}_{n}) + \vec{c}_{n} $$

with

6 $$ R = \left( \begin{array}{ccc} \cos\Delta\phi & -\sin\Delta\phi & 0\\[6 pt] \sin\Delta\phi & \cos\Delta\phi & 0\\[6 pt] 0 & 0 & 1\\ \end{array}\right)$$

as the implemented knee joint has only one rotational degree of freedom in the sagittal plane (z-axis of the femur frame) and

7 $$ \vec{c}_{n} = \frac{\vec{c}^*_{n} + \vec{c}^*_{n-1}}{2}$$

where $\vec{c}^*_{n}$ and $\vec{c}^*_{n-1}$ are the ICOR for the current and previous time step, respectively. $n$ is the current time step, $\vec{b}_{n}$ and $\vec{b}_{n-1}$ are the position of the tibia frame in the femur frame for the current and previous timestep, respectively. $\Delta\phi$ is the change in angle between the previous and current time step ($\phi_{n} - \phi_{n-1}$) and $\vec{c}_{n}$ is defined as the mean between the ICOR of the current ($\vec{c}^*_{n}$) and previous ($\vec{c}^*_{n-1}$) time step.

## Results

### Double pendulum model

#### Estimation of joint center of rotation with and without added noise

**Fig. 6:**
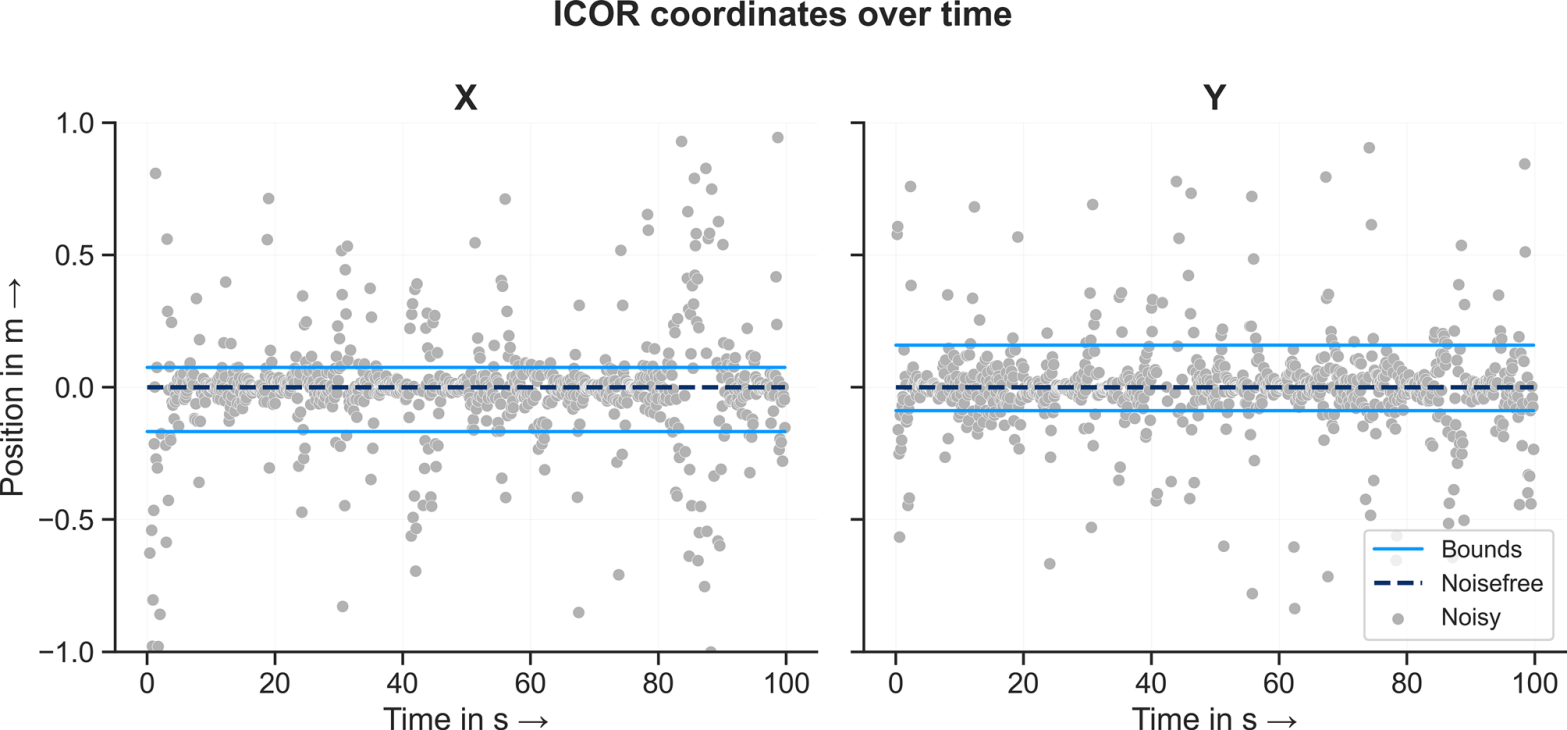
Coordinates of the ICOR as a function of time for the double pendulum model. For each coordinate, the light blue lines indicate the upper and lower bounds used to determine the outliers which are defined by being outside the 1.5-fold interquartile range. The dark blue dashed line shows the calculated values for the noisefree data which is the known position of the ICOR.

For each time step of the forward simulation, corresponding to 1000 data points, the ICOR between both bodies of the double pendulum model was computed using both noisefree and noisy input data. For both conditions, the resulting coordinates of the ICOR as a function of time are shown in Fig. 6. For the noisefree data, the ICOR coordinate values are 0 for every timestep. To enhance the results for the noisy data, outliers were defined and excluded from the computed ICOR values; 635 data points remained. For both coordinates, the values of the ICOR coordinates scatter widely and evenly around the true value 0 for the noisy data. Minimum and maximum values were $-187.54 \, m$ to $5.81 \, m$ and $-51.06 \ m$ to $41.71 \, m$ for the x- and y-coordinate respectively. Single values deviate quite far from the majority of the data points. The axis limits of Fig. 6 have been restricted to the interval $[-1,1]$ in order to visualize the majority of the data. The 1.5-fold interquartil distance was used to define outliers for each coordinate. The resulting cutoff values were $x_{upper} = 0.0756 \, m$, $x_{lower} = -0.1673 \, m$, $y_{upper} = 0.1597 \, m$ and $y_{lower} = -0.0880 \, m$. Values outside these ranges have been removed to enhance the quality of the ICOR computation results.

**Fig. 7:**
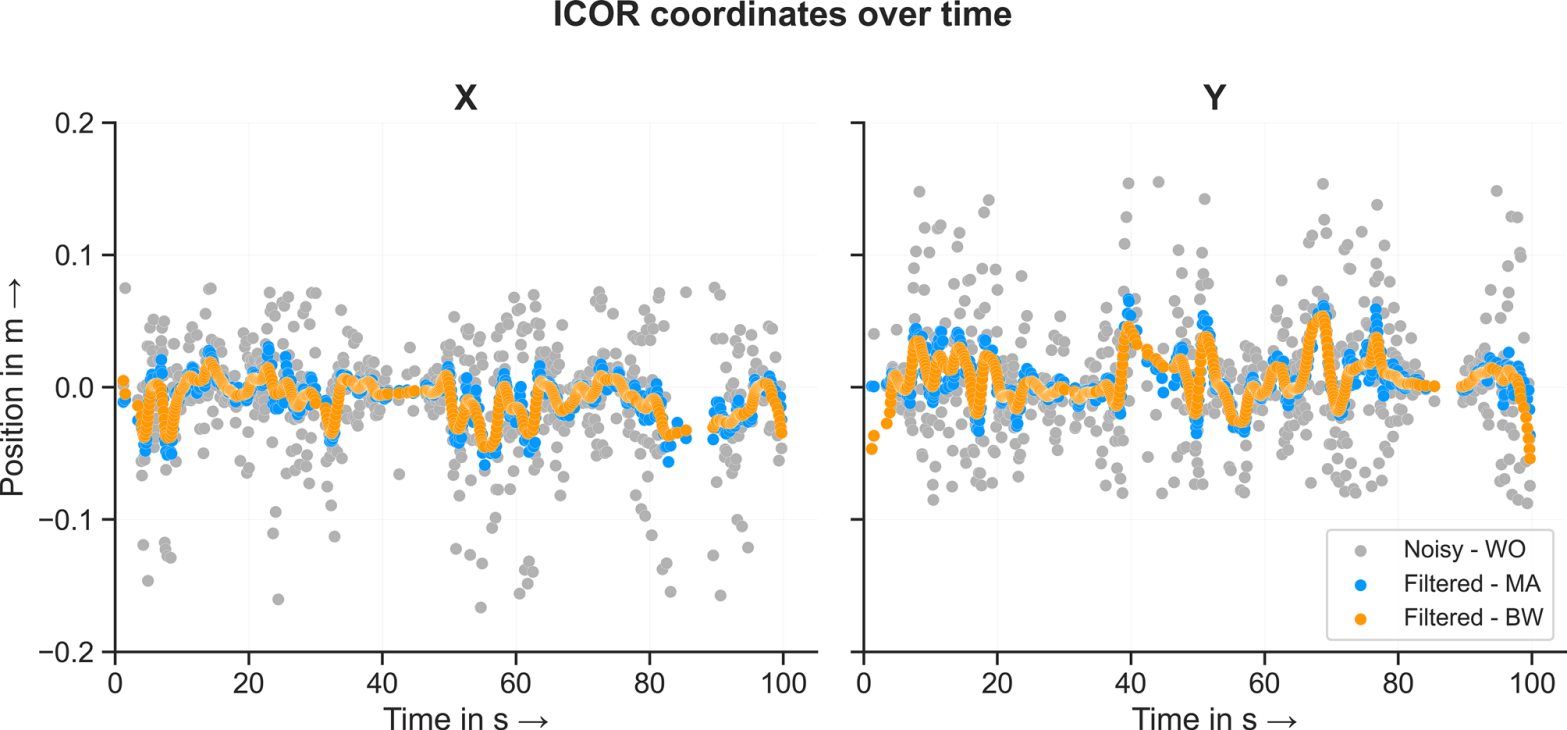
Coordinates of the ICOR as a function of time for the double pendulum model: unfiltered without outliers and filtered using either a moving average or a Butterworth filter

To further compensate the effect of the noisy input data, the computed coordinates of the ICOR as a function of time were filtered using either a moving average or a Butterworth filter, see Fig. 7. Both filters decrease the amplitude of the ICOR coordinate values in comparison to the noisy data wihout outliers (WO). When comparing the results of the two filters, it is noticeable that the Butterworth filter generally produces a smoother data curve. However, the general course of the two curves is quite similiar. Both filters decrease the amplitude of the noisy data. The lower amplitude of the data points further enhances the quality of the ICOR computation results.

**Fig. 8:**
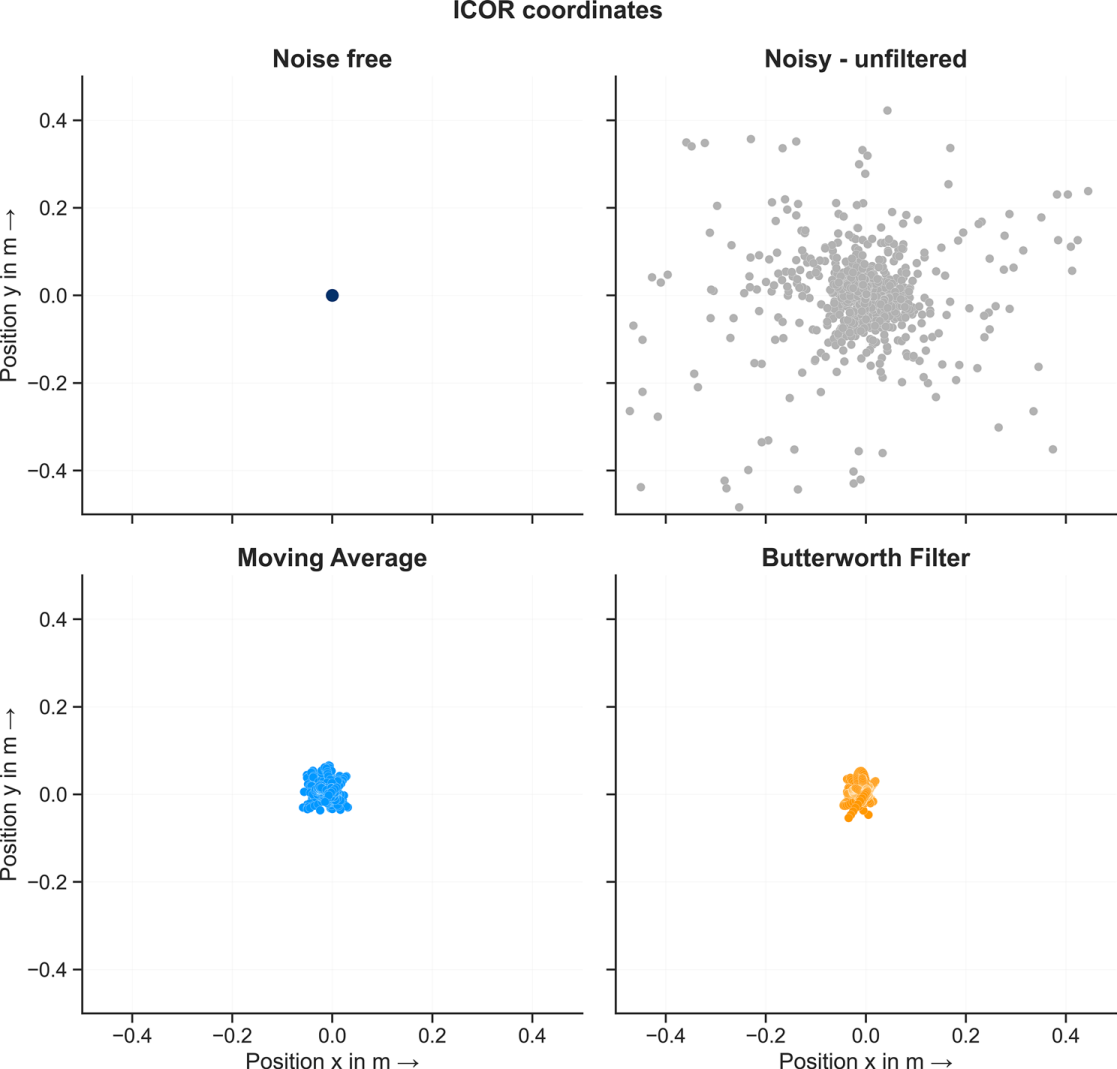
Coordinates of the ICOR for the double pendulum model for every condition: noisefree, noisy, IAOR coordinates filtered using a moving average and IAOR coordinates filtered using a Butterworth filter

Next, we analyzed the effect of removing the outliers and filtering the coordinates over time on the computed ICOR results. The coordinates of the ICOR for each time step and condition: noisefree, noisy, filtered - MA and filterd - BW are shown in Fig. 8. For the noisefree condition, the method computes the true value of the joint center at $P(0/0)$ for every time step. For the noisy unfiltered condition, the coordinates of the ICOR scatter evenly around the true value $P$. Removing outliers and filtering the data leads to the coordinates scattering evenly around the true value with a lower amplitude in comparison to the unfiltered condition. This applies for both filtering methods. The mean, median and RMSE for every condition are listed in Table 1. In comparison to the unfiltered mean values, both filtering methods decrease the deviation from the known joint center position. In contrast, the median of the filtered data is slightly larger than the noisy unfiltered data. This is probably due to the fact that only white Gaussian noise was added to the data. As a result, the noisy data scatters evenly around the true value and the median allows it to be identified very well even without filtering the data. Both filtering methods decrease the RMSE between the true and estimated joint position to about the same level. Removing outliers and filtering the noisy input data makes it possible to identify the position of the joint center of a simple revolute joint.

**Table 1:** Mean, median and rmse (in m) of computed ICOR results for every condition

	Mean		Median		RMSE	
	x	y	x	y	x	y
Noisefree	0.0000	0.0000	0.0000	0.0000	0.0000	0.0000
Noisy data	−0.2900	−0.0684	−0.0014	−0.0023	6.1280	2.4097
MA-WO^1^	−0.0076	0.0059	−0.0039	0.0027	0.0168	0.0182
BW-WO^2^	−0.0077	0.0055	−0.0048	0.0030	0.0150	0.0163

^1^ MA-WO: Moving average filter without outliers, ^2^ BW-WO: Butterworth filter without outliers

### Musculoskeletal model example

#### Estimation of hip joint center of rotation for both noisefree and noisy input data

**Fig. 9:**
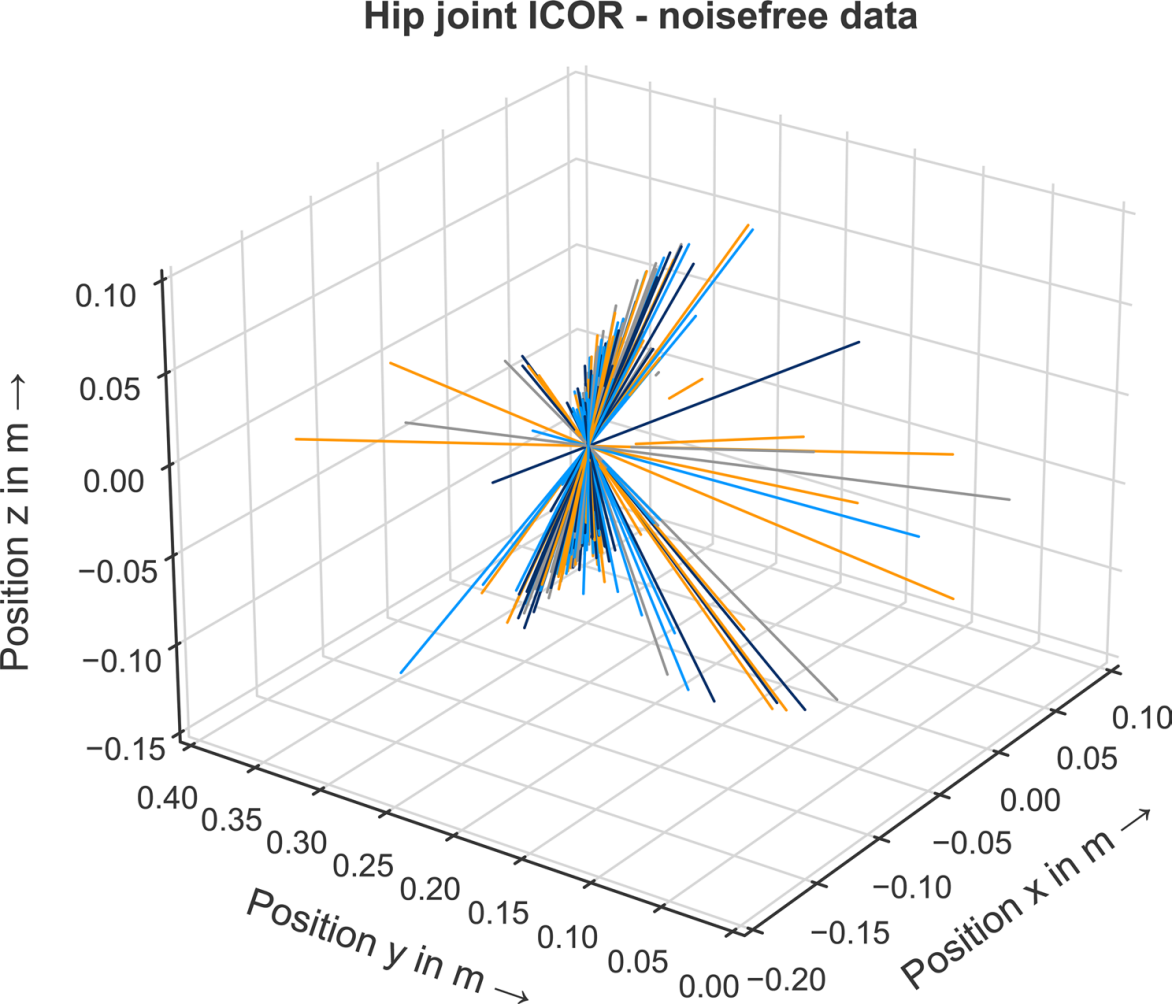
Hip joint IAOR for the noisefree data for each time step. All lines intersect exactly in one point which depicts the ICOR of the hip joint

To verify that the ICOR identification method can correctly identify three-dimensional joints of musculoskeletal models, we first computed the ICOR of the hip joint for the noisefree data for each of the 969 data points. The IAORs for every time step intersect in exactly one point: $P_{intersect} = (-0.05/ 0.25/-0.02)$, see Fig. 9. The hip joint of the musculoskeletal model is defined in the origin of the femur frame. The computed intersection point corresponds exactly to the position of the virtual femur IMU frame in the femur frame which defines the position of the hip joint in the IMU femur frame. The method is able to correctly identify the center of rotation of a ball joint when noisefree synthetic data is used.

**Fig. 10:**
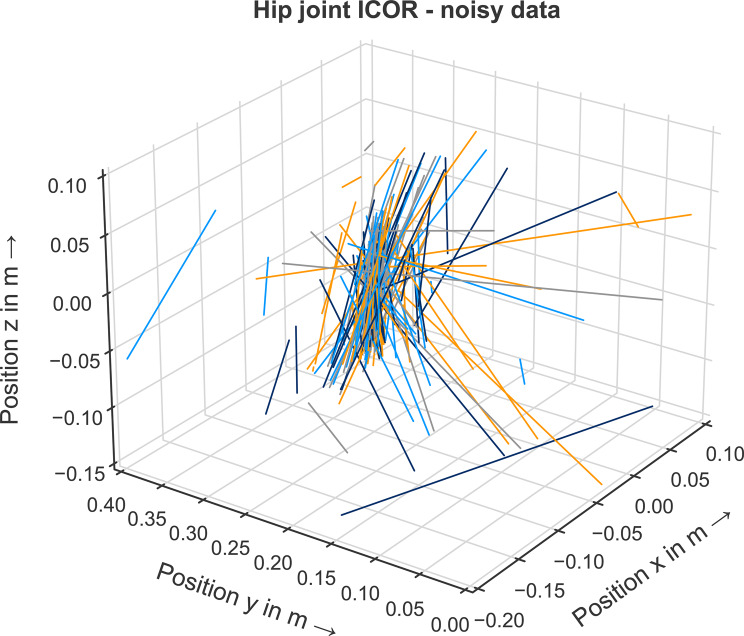
Hip joint IAOR for the noisy data for each time step. No point of intersection between the lines is visually identifiable

Next, we analyzed whether it is possible to estimate the hip joint center of rotation from noisy input data. For the noisy data, no single point of intersection of all IAORs is visually identifiable, see Fig. 10. However, the calculated mean point of intersection is $$P_{intersect}^{noisy}$$$$= (-0.052/0.2505/$$$$-0.0215)$$. This result corresponds well with the known joint center position as deviations for the x-, y- and z-coordinates are in the millimeter range. Using an optimization approach to compensate the noisy input data, the method is able to estimate the center of rotation of the hip joint.

#### Estimation of varying knee joint axis for both noisefree and noisy input data

To determine whether the method is able to correctly identify a moving center of rotation – dependening on the angle of the joint – we investigated the knee joint of the musculoskeletal model. Simm spline functions define the position of the tibia frame in relation to the femur frame depending on the knee angle in the sagittal plane. The functions are defined by data points, which are used to create smooth functions using cubic splines. These functions can be calculated based on the ICOR.

**Fig. 11:**
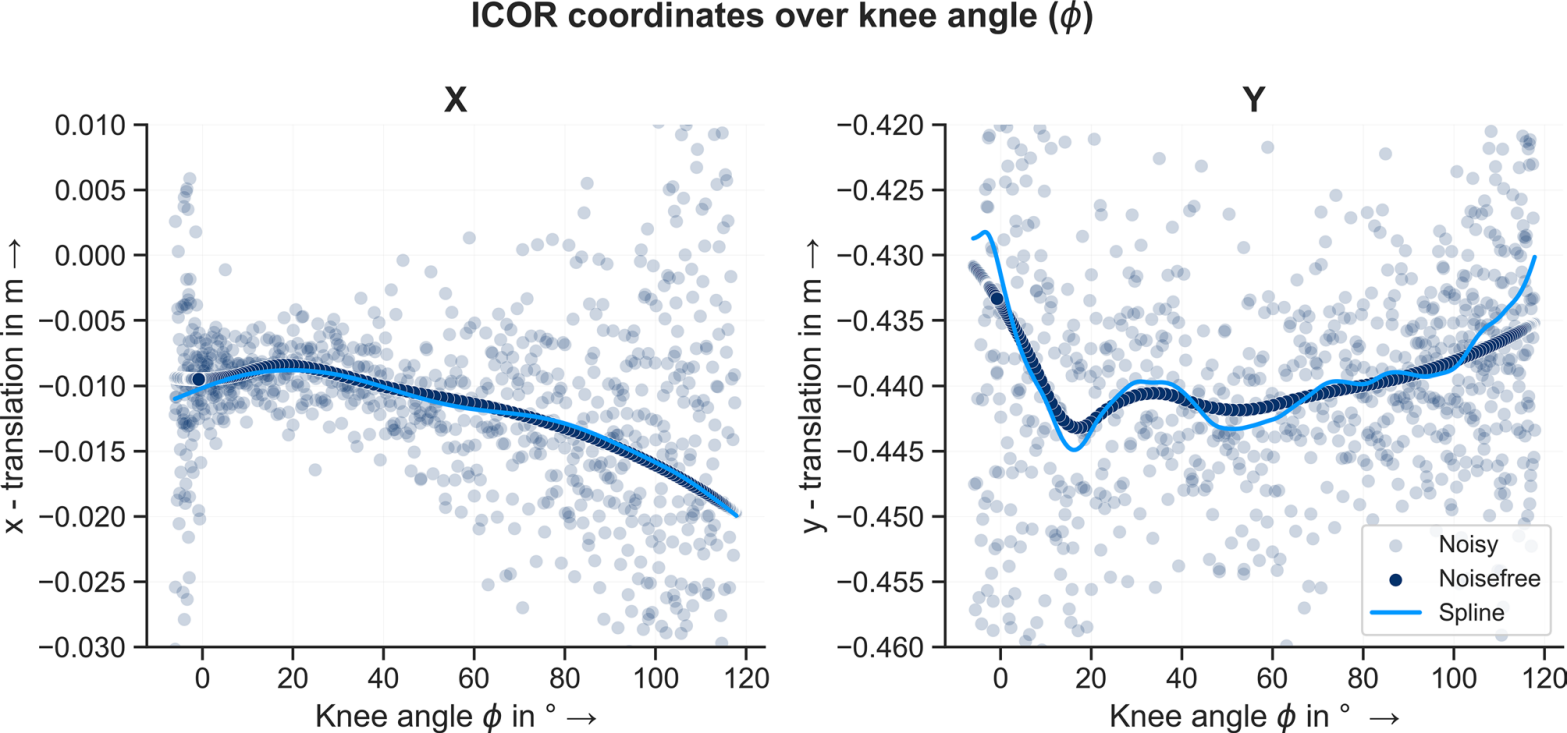
ICOR coordinates over knee angle $\phi$ for noisefree and noisy input data. The light blue lines show resulting smoothing splines for the noisy data. For the x-coordinate, the spline corresponds very well to the noisefree data. For the y-coordinate, greater deviations are observable

**Fig. 12:**
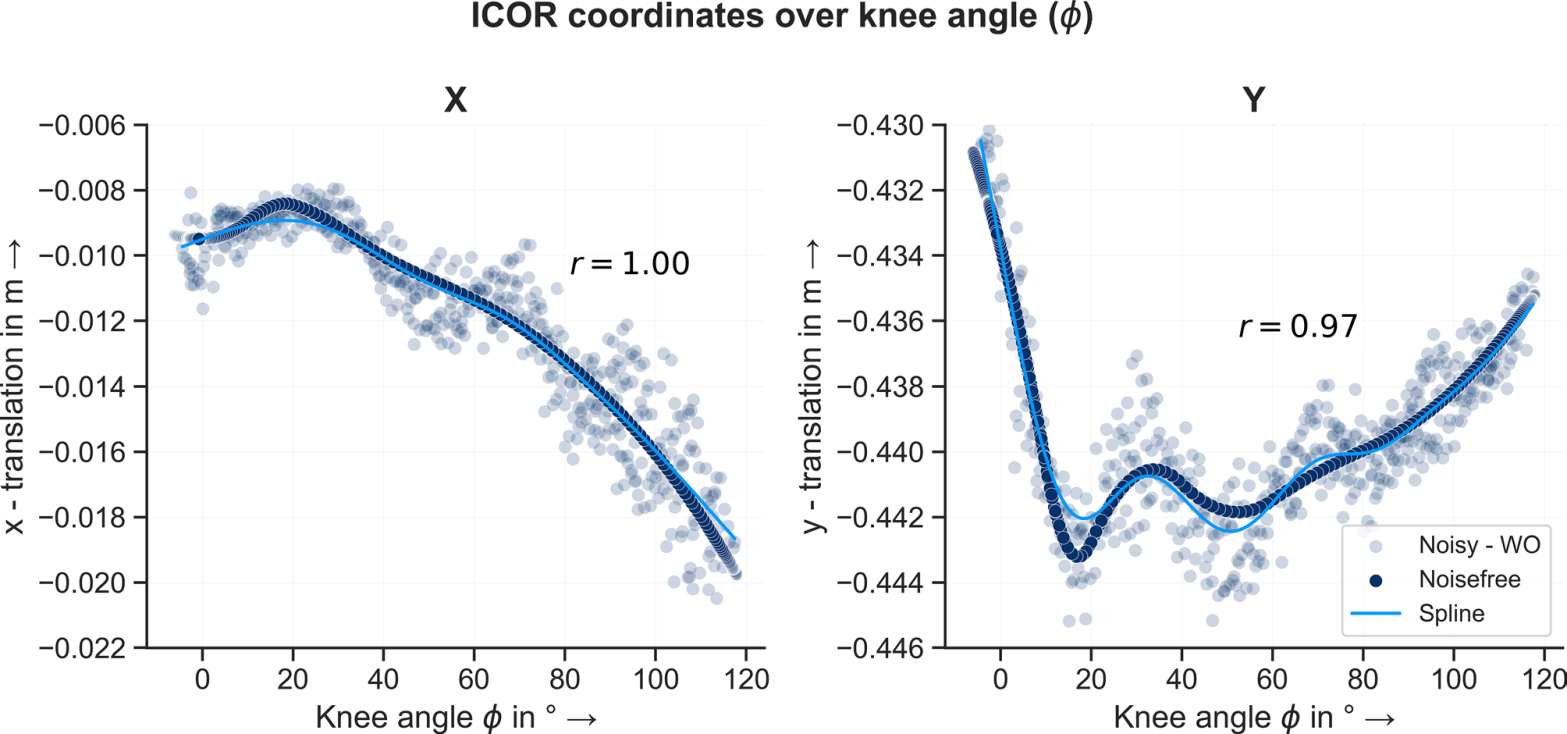
ICOR coordinates over knee angle $\phi$ for noisefree and noisy data without outliers. For both coordinates the course of the curves match very well

The results for the ICOR position coordinates (x,y) both for the noisefree and noisy data for each of the 969 data points as a function of the knee angle $\phi$ are visualized in Fig. 11. For the noisy input data, the ICOR coordinate values scatter widely around the curve calculated using the noisefree data. Prior to data smoothing, certain data points were excluded to mitigate their adverse effects on the smoothing results as they deviated significantly from the majority of the data. Minimum and maximum values were then $-2.83 m$, $1.16 \, m$ and $-4.07 \, m$, $1.86 \, m$ for the x- and y-coordinate respectively. For the x-coordinate, the amplitude of the scatter is highest for negative knee angle values (knee extension). The amplitude then decreases at $0^{\circ}$ flexion and increases again with increasing flexion angle. For the y-coordinate, the noisy data scatters the most for negative knee angles and very high flexion angles. Between $20^{\circ}$ and $100^{\circ}$ flexion, the noisy data scatters evenly around the noisefree data. The noisy data was smoothed using the smoothing spline function of Matlab 2021a [43], see Fig. 12. The smoothing parameters for the x- and y-coordinate were $3e-5$ and $0.003$ respectively. For the x-coordinate, the spline corresponds very well to the noisefree data. For the y-coordinate, greater deviations are visible between the noisefree curve and the spline. This is attributable to the more complex curve of the y-coordinate in comparison to the x-coordinate.

The splines are used to define outliers of the noisy data. A margin of values surrounding the splines is defined $(\pm \, 1 \, cm)$ and values of the ICOR coordinates outside this range are defined as outliers and removed. A total of 319 data points were removed. Afterwards, the data was filtered using either a moving average or a Butterworth filter. The filtered data was again smoothed using the smoothing spline function. The results for the data filtered using the moving average are shown in Fig. 12. The figures for the data filtered using a Butterworth filter are listed in the supplementary material. To quantify the goodness of the fit, the correlation coefficients between the noisefree data and the smoothing splines were calculated for both applied filters and coordinates. They are listed in Table 2. For both coordinates, the smoothing splines correspond very well to the noisy data and the coefficients of correlation are high.

**Table 2:** Coefficients of correlation between true and smoothed ICOR data for each applied filter and coordinate

	x	y
MA-WO^1^	0.9954	0.9742
BW-WO^2^	0.9949	0.9712

^1^MA-WO: Moving average filter without outliers

^2^BW-WO: Butterworth filter without outliers

**Fig. 13:**
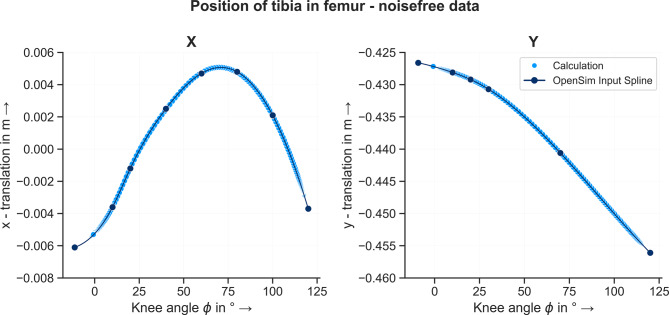
Position of tibia frame in femur frame over knee angle based on noisefree data. The dark blue dots show the data points of the spline function which defines the movement of the tibia in the femur frame depending on the knee angle. The light blue dots show the calculated position of the tibia based on the identified joint axis

**Fig. 14:**
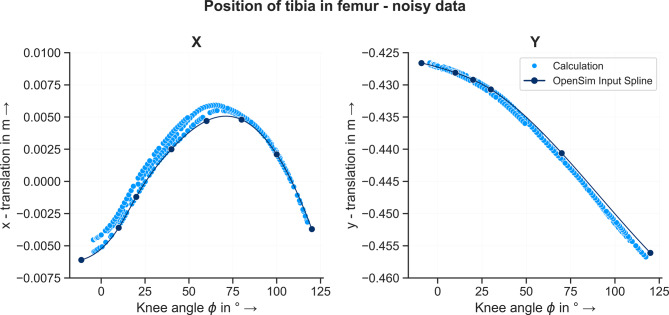
Position of tibia in femur frame over knee angle based on noisy data, filtered using the moving average method. The dark blue dots show the data points of the spline function which defines the movement of the tibia in the femur frame depending on the knee angle. The light blue dots show the calculated position of the tibia based on the identified joint axis

Based on the identified knee joint axes the position of the tibia frame in relation to the femur frame was computed using the approach described in Section “Process description”. Starting from the known location of the tibia in the femur frame at $\phi = 0^{\circ}$, the new position is calculated consecutively for every time step. The resulting curve of the tibia position for both the noisefree and noisy/filtered - MA condition is shown in Figs. 13 and 14. The RMSEs between the known and calculated position of the tibia in the femur frame are listed in Table 3. For the noisefree input data, the calculated position of the tibia as a function of the knee angle matches the data points which define the knee joint in the model. The RMSE for both coordinates is zero. This means that the curve of the ICOR over $\phi$ calculated with the noisefree data describes the moving center of rotation of the knee joint. For the noisy input data, small deviations between the calculated position of the tibia and the data points are observable. However, for both coordinates and filters, the RMSEs are equal to or smaller than $1 \, mm$. Using smoothing splines and filtering methods to compensate the noisy input data, the method is able to estimate the moving center of rotation – depending on the knee angle – of the knee joint and based on the identified ICOR, the position of the tibia in the femur frame can be calculated.

**Table 3:** RMSEs (in m) between OpenSim input spline and calculated position of tibia in femur

	x	y
noisefree	0.0000	0.0000
MA-WO^1^	0.0007	0.0008
BW-WO^2^	0.0008	0.0010

^1^MA-WO: Moving average filter without outliers

^2^BW-WO: Butterworth filter without outliers

## Discussion

In this article we have shown the feasibility and performance of a direct, analytical method for identifying the fixed and moving centers of rotation of arbitrary joints from virtual noisy motion data in musculoskeletal models in OpenSim. The centers of rotation of various joints were calculated using noisefree synthetic data and compared to the implemenation of the respective joints in the model. Following this verification, white Gaussian noise was added to the motion data to analyze the performance of the method for identifiying arbitrary joints based on noisy input data. Using the method, we were able to identify joint centers or axes of rotations with high accuracy ($$ RMSE < 0.01$$ and $$r=0.97 - 0.99$$) for three different joints of ranging complexity implemented in OpenSim: a planar revolute joint, a ball joint (hip joint) and a complex custom joint which uses input functions to define the movement of the child frame in the parent frame and thus defines the joint (knee joint).

Even though we were able to identify joint axes or centers of rotation based on synthetic noisy input data, there are also limitations regarding the results. One limitation of this simulation study is that only virtual and no experimentally measured motion data has been used to analyze the performance of the analytical method for identifying fixed and moving centers of rotation of arbitrary joints in OpenSim. It is difficult to infer how well we will be able to identify the IAOR or ICOR from experimental motion data. However, we chose using virtual motion data, as the main purpose of this article was to demonstrate the general applicability of the analytical method for analyzing and identifying arbitrary joints in OpenSim based on motion data as the principle of the ICOR has not yet been applied in biomechanical simulations. Using virtual motion data that were created with a known model enabled us to verify the method as the ground truth was known (defined joint centers and input spline functions). In the next step, we want to identify individualized joint centers or axes of rotations based on experimentally measured motion data.

Another limitation is that the noisy input data was generated by adding only white Gaussian noise to the virtual motion data. This is not an accurate representation of experimentally measured motion data as the data is subject to further error sources. Especially IMUs are not only subject to measurement noise. One of the major issues with IMUs is that they are subject to sensor drift. In the presented method, this error would affect the quality of the vector that indicates the position of body 2 (the child segment) in reference to body 1 (the parent segment). A drift in this vector would mean that the same drift will be present in the calculated IAOR or ICOR. However, as it is difficult to correctly model the measurement error of an IMU, as most IMU systems include system-inherent error reduction and compensation algorithms, we decided to add only white Gaussian noise. Therefore, experimental motion data will have a lower accuracy than the noisy virtual data used in this simulation study. It is necessary to determine the extent to which the desired fixed and moving joint centers can be accurately calculated from input data that contains errors. If the errors in the experimental measurement data are too large to obtain accurate results, one possible solution could be to develop strategies for enhancing the accuracy of the input data. Previous research has shown that fusing IMU data with either RGB- [44, 45] or depth-camera [46] data can enhance motion tracking performance in comparison to single modality approaches. So, using multimodal motion data may enhance the method’s robustness by mitigating measurement errors inherent to one sensor type (IMU drift) through complementary data acquisition from another sensor type (e.g. RGB- or depth-camera), which may lead to input data with better accuracy. Another solution may be to identify the most appropriate movement patterns as well as their range of motion and duration for identifying ICORs. For the method presented by Ehrig et al. [30] to determine the center of rotation of the hip joint, a combination of several flexion-extension and abduction-adduction movements of the thigh was determined to generate the best possible estimate [47]. Similar investigations could also enhance the results of the approach presented in this paper.

The combination of the lack of experimental data and oversimplified noise modelling represents the main limitation, restricting real-world applicability. Prior to clinical or biomechanical application, it is essential to validate against experimental IMU/motion capture datasets.

One advantage of the presented approach is that it is suitable for arbitrary joints based on arbitrary motions in OpenSim. The method is not constrained by the type of joint to be analyzed or the number of bodies in motion. As shown in Section “Results”, we were able to identify joint centers of rotation based on noisy data for simple, two-dimensional joints (revolute joint) but also more complex joints like the three dimensional hip joint (ball joint) and the knee joint (custom joint) with a moving center of rotation. In addition, it does not matter whether only one body or both joint partners, parent and child segment, undergo motion. Most approaches in this area cannot identify the joint axes when both joint partners are in motion (sphere-fit methods [26, 28, 29, 48, 49], one-sided transformation techniques [50–52], IMU-based method for fixed axes [36]), or if they can, they are not applicable to arbitrary joint types (two-sided transformation techniques [53], IMU-based approaches for moving axes [37]). The investigated approach distinguishes itself from previously published work by adressing these two aspects. As long as all required input data is available, this approach can be used without any restrictions, regardless of the joint type or number of bodies in motion.

Further, although our method is based on IMU data it can, in principle, be applied to data from any measurement system that generates the necessary input data. A wide range of possible measurement systems is now available for recording experimental motion data (marker-based, IMU-based, depth camera-based), including multimodal approaches (IMU and RGB- or depth-camera). Thus, the method is not only independent of the type of joint and motion but also of the measurement system used for gathering the experimental motion data. A novel approach in the realm of motion capturing for musculoskeletal simulations involves the utilization of radar sensors [54]. Radars can measure velocities with high precision which makes them particulary compelling for the method presented in this article. Enhanced precision in the input data of the method leads to less dependence on filtering methods and thereby contributes to an enhanced reliability and accuracy in the computed results. This makes the method universally applicable for current and probably future measurement systems.

## Conclusion

We have shown the feasibility and performance of an analytical method for identifying both fixed and moving joint centers of rotation of arbitrary joints from virtual noisy motion data in musculoskeletal models in OpenSim. We were able to identify joint centers or axes of rotations with satisfying accuracy for three different joints of ranging complexity. Future research will determine the performance of the method for identifying instantaneous axes or centers of rotation from experimental motion data. Using this approach we, eventually, aim to individualize joint centers and axes of rotations to achieve more accurate motion tracking results. We expect that the higher the consistency between a person and the corresponding model is, the more reliable kinematic and dynamic computed results will be. In general, the better the musculoskeletal model represents the measured person, the better experimental data can be tracked. Better tracking performances leads to enhanced kinematic consistency which is a prerequisite for getting reliable dynamic simulation results (e.g. joint moments or joint reaction forces). As we were able to identify joint centers and axes of rotation based on virtual noisy motion data we expect to be able to personalize joint centers and axes of rotation based on experimental measurements to enhance kinematic and dynamic simulation results. Eventually, future research could focus on identifying healthy and pathological joint axes and centers of rotation and comparing them.

## Electronic supplementary material

Below is the link to the electronic supplementary material.

Supplementary Material 1

Supplementary Material 2

Supplementary Material 3

## Data Availability

The datasets used and/or analyzed during the current study are available from the corresponding author on reasonable request.

## References

[1] Kainz H, Koller W, Wallnöfer E, Bader TR, Mindler GT, Kranzl A. A framework based on subject-specific musculoskeletal models and Monte Carlo simulations to personalize muscle coordination retraining. Sci Rep. 2024;14(1):3567. 10.1038/s41598-024-53857-9. 38347085 10.1038/s41598-024-53857-9 PMC10861532

[2] Meireles S, Wesseling M, Smith CR, Thelen DG, Verschueren S, Jonkers I. Medial knee loading is altered in subjects with early osteoarthritis during gait but not during step-up-and-over task. PLoS One. 2017;12(11):0187583. 10.1371/journal.pone.0187583. 10.1371/journal.pone.0187583 PMC5678707 29117248

[3] Pellikaan P, Giarmatzis G, Vander Sloten J, Verschueren S, Jonkers I. Ranking of osteogenic potential of physical exercises in postmenopausal women based on femoral neck strains. PLoS One. 2018;13(4):0195463. 10.1371/journal.pone.0195463. 10.1371/journal.pone.0195463 PMC5884624 29617448

[4] Caprara S, Moschini G, Snedeker JG, Farshad M, Senteler M. Spinal sagittal alignment goals based on statistical modelling and musculoskeletal simulations. J Biomech. 2020;102:109621. 10.1016/j.jbiomech.2020.109621. 31959392 10.1016/j.jbiomech.2020.109621

[5] Guo J, Wang J, Chen J, Ren G, Tian Q, Guo C. Multibody dynamics modeling of human mandibular musculoskeletal system and its applications in surgical planning. Multibody Syst Dyn. 2023;57(3–4):299–325. 10.1007/s11044-023-09876-x.

[6] Watters TS, Mather RC, Browne JA, Berend KR, Lombardi AV, Bolognesi MP. Analysis of procedure-related costs and proposed benefits of using patient-specific approach in total knee arthroplasty. J Surg Orthop Adv. 2011;20(2):112–16. 21838072

[7] Ackland DC, Robinson D, Redhead M, Lee PVS, Moskaljuk A, Dimitroulis G. A personalized 3D-printed prosthetic joint replacement for the human temporomandibular joint: from implant design to implantation. J Mech Behav Biomed Mater. 2017;69:404–11. 10.1016/j.jmbbm.2017.01.048. 28199931 10.1016/j.jmbbm.2017.01.048

[8] Dérand P, Rännar L-E, Hirsch J-M. Imaging, virtual planning, design, and production of patient-specific implants and clinical validation in craniomaxillofacial surgery. Craniomaxillofac Trauma Reconstr. 2012;5(3):137–43. 10.1055/s-0032-1313357 23997858 10.1055/s-0032-1313357 PMC3578652

[9] Van Wouwe T, Hicks J, Delp S, Liu KC. A simulation framework to determine optimal strength training and musculoskeletal geometry for sprinting and distance running. PLoS Comput Biol. 2024;20(2):1011410. 10.1371/journal.pcbi.1011410. 10.1371/journal.pcbi.1011410 PMC10917303 38394308

[10] McErlain-Naylor SA, King MA, Felton PJ. A review of forward-dynamics simulation models for predicting optimal technique in maximal effort sporting movements. Appl Sci. 2021;11(4):1450. 10.3390/app11041450.

[11] Rasmussen J, Boocock M, Paul G. Advanced musculoskeletal simulation as an ergonomic design method. Work. 2012;41:6107–11. 10.3233/WOR-2012-1069-6107.

[12] Jeang A, Chiang AJ, Chiang PC, Chiang PS, Tung PY. Robust parameters determination for ergonomical product design via computer musculoskeletal modeling and multi-objective optimization. Comput Ind Eng. 2018;118:180–201. 10.1016/j.cie.2018.02.013.

[13] Lu TW, O’Connor JJ. Bone position estimation from skin marker coordinates using global optimisation with joint constraints. J Biomech. 1999;32(2):129–34. 10.1016/s0021-9290(98)00158-4 10052917

[14] Al Borno M, O’Day J, Ibarra V, Dunne J, Seth A, Habib A, et al. OpenSense: an open-source toolbox for inertialmeasurement-unit-based measurement of lower extremity kinematics over long durations. J Educ Chang Neuroeng And Rehabil. 2022;19(1):22. 10.1186/s12984-022-01001-x. 10.1186/s12984-022-01001-x PMC8859896 35184727

[15] Tagliapietra L, Modenese L, Ceseracciu E, Mazzà C, Reggiani M. Validation of a model-based inverse kinematics approach based on wearable inertial sensors. Comput Methods Biomech Biomed engin. 2018;21(16):834–44. 10.1080/10255842.2018.1522532. 30466324 10.1080/10255842.2018.1522532

[16] Wechsler I, Wolf A, Fleischmann S, Waibel J, Molz C, Scherb D, et al. Method for using IMU-Based experimental motion data in BVH format for musculoskeletal simulations via OpenSim. Sensors. 2023;23(12):5423. 10.3390/s23125423. 37420590 10.3390/s23125423 PMC10303752

[17] Lund ME, Andersen MS, Zee M, Rasmussen J. Scaling of musculoskeletal models from static and dynamic trials. Int Biomech. 2015;2(1):1–11. 10.1080/23335432.2014.993706.

[18] Seth A, Hicks JL, Uchida TK, Habib A, Dembia CL, Dunne JJ, Ong CF, DeMers MS, Rajagopal A, Millard M, Hamner SR, Arnold EM, Yong JR, Lakshmikanth SK, Sherman MA, Ku JP, Delp SL. OpenSim: simulating musculoskeletal dynamics and neuromuscular control to study human and animal movement. PLoS Comput Biol. 2018;14(7):1006223. 10.1371/journal.pcbi.1006223 10.1371/journal.pcbi.1006223 PMC6061994 30048444

[19] Camomilla V, Cereatti A, Cutti AG, Fantozzi S, Stagni R, Vannozzi G. Methodological factors affecting joint moments estimation in clinical gait analysis: a systematic review. Biomed Eng Online. 2017;16(1):106. 10.1186/s12938-017-0396-x. 28821242 10.1186/s12938-017-0396-x PMC5563001

[20] Bartels W, Demol J, Gelaude F, Jonkers I, Vander Sloten J. Computed tomography-based joint locations affect calculation of joint moments during gait when compared to scaling approaches. Comput Methods Biomech Biomed engin. 2015;18(11):1238–51. 10.1080/10255842.2014.890186. 24641349 10.1080/10255842.2014.890186

[21] Lenaerts G, Bartels W, Gelaude F, Mulier M, Spaepen A, Van Der Perre G, Jonkers I. Subject-specific hip geometry and hip joint centre location affects calculated contact forces at the hip during gait. J Biomech. 2009;42(9):1246–51. 10.1016/j.jbiomech.2009.03.037. 19464012 10.1016/j.jbiomech.2009.03.037

[22] Reinbolt JA, Haftka RT, Chmielewski TL, Fregly BJ. Are patient-specific joint and inertial parameters necessary for accurate inverse dynamics analyses of gait? IEEE Trans Biomed Eng. 2007;54(5):782–93. 10.1109/TBME.2006.889187. 17518274 10.1109/TBME.2006.889187 PMC3608472

[23] Stagni R, Leardini A, Cappozzo A, Grazia Benedetti M, Cappello A. Effects of hip joint centre mislocation on gait analysis results. J Biomech. 2000;33(11):1479–87. 10.1016/S0021-9290(00)00093-2. 10940407 10.1016/s0021-9290(00)00093-2

[24] Holden JP, Stanhope SJ. The effect of variation in knee center location estimates on net knee joint moments. Gait & Posture. 1998;7(1):1–6. 10.1016/S0966-6362(97)00026-X. 10200370 10.1016/s0966-6362(97)00026-x

[25] Most E, Axe J, Rubash H, Li G. Sensitivity of the knee joint kinematics calculation to selection of flexion axes. J Biomech. 2004;37(11):1743–48. 10.1016/j.jbiomech.2004.01.025. 15388317 10.1016/j.jbiomech.2004.01.025

[26] Bell AL, Pedersen DR, Brand RA. A comparison of the accuracy of several hip center location prediction methods. J Biomech. 1990;23(6):617–21. 10.1016/0021-9290(90)90054-7. 2341423 10.1016/0021-9290(90)90054-7

[27] Leardini A, Cappozzo A, Catani F, Toksvig-Larsen S, Petitto A, Sforza V, Cassanelli G, Giannini S. Validation of a functional method for the estimation of hip joint centre location. J Biomech. 1999;32(1):99–103. 10.1016/S0021-9290(98)00148-1. 10050957 10.1016/s0021-9290(98)00148-1

[28] Piazza SJ, Okita N, Cavanagh PR. Accuracy of the functional method of hip joint center location: effects of limited motion and varied implementation. J Biomech. 2001;34(7):967–73. 10.1016/S0021-9290(01)00052-5. 11410180 10.1016/s0021-9290(01)00052-5

[29] Cappozzo A. Gait analysis methodology. Hum Mov Sci. 1984;3(1–2):27–50. 10.1016/0167-9457(84)90004-6.

[30] Ehrig RM, Taylor WR, Duda GN, Heller, MO. A survey of formal methods for determining the centre of rotation of ball joints. J Biomech. 2006;39(15):2798–809. 10.1016/j.jbiomech.2005.10.002. 16293257 10.1016/j.jbiomech.2005.10.002

[31] Roetenberg D, Luinge HJ, Slycke P. Xsens MVN: full 6DOF human motion tracking using miniature inertial sensors. 2013. http://www.xsens.com.

[32] Luinge HJ, Veltink PH. Measuring orientation of human body segments using miniature gyroscopes and accelerometers. Med Biol Eng Comput. 2005;43(2):273–82. 10.1007/BF02345966. 15865139 10.1007/BF02345966

[33] Zhang J-T, Novak AC, Brouwer B, Li Q. Concurrent validation of xsens MVN measurement of lower limb joint angular kinematics. Physiol Meas. 2013;34(8):63–69. 10.1088/0967-3334/34/8/N63. 10.1088/0967-3334/34/8/N63 23893094

[34] Caputo F, Greco A, D’Amato E, Notaro I, Spada S. IMU-based motion capture wearable system for ergonomic assessment in industrial environment. In: Ahram TZ, editor. Advances in Human Factors in Wearable Technologies and Game Design. Cham: Springer; 2019. p. 215–25. (Advances in Intelligent Systems and Computing; vol. 795). http://link.springer.com/10.1007/978-3-319-94619-1_21.

[35] Narváez F, Árbito F, Proaño R. A quaternion-based method to IMU-to-body alignment for gait analysis. In: Duffy VG, editor. Digital Human Modeling: Applications in Health, Safety, Ergonomics, and Risk Management. Cham: Springer; 2018. p. 217–31. (Lecture Notes in Computer Science; vol. 10917). https://link.springer.com/10.1007/978-3-319-91397-1_19.

[36] Garcia-de-Villa S, Jimenez-Martin A, Garcia-Dominguez JJ. Novel IMUBased adaptive estimator of the Center of rotation of joints for movement analysis. IEEE Trans Instrum Meas. 2021;70:1–11. 10.1109/TIM.2021.3073688.33776080

[37] Olsson F, Halvorsen K. Experimental evaluation of joint position estimation using inertial sensors. In: 2017 20th International Conference on Information Fusion (Fusion). Xi’an, China: IEEE; 2017, pp. 1–8. http://ieeexplore.ieee.org/document/8009669/.

[38] Walker PS, Rovick JS, Robertson DD. The effects of knee brace hinge design and placement on joint mechanics. J Biomech. 1988;21(11):965–74. 10.1016/0021-9290(88)90135-2. 3253283 10.1016/0021-9290(88)90135-2

[39] Gross D, Hauger W, Schnell W, Schröder J, Wall WA. Technische Mechanik. Band 3: Kinetik. 9th ed. Berlin, Heidelberg: Springer; 2006. 10.1007/3-540-34085-8.

[40] Kunz DL. Instantaneous center/axis of rotation for planar and threedimensional motion. Int J Mech Eng Educ. 2022;50(3):692–703. 10.1177/03064190211051104.

[41] Miehling J. Musculoskeletal modeling of user groups for virtual product and process development. Comput Methods Biomech Biomed engin. 2019;22(15):1209–18. 10.1080/10255842.2019.1651296. 31401869 10.1080/10255842.2019.1651296

[42] Scherb D, Steck P, Wechsler I, Wartzack S, Miehling J. The determination of assistance-as-needed support by an ankle–foot orthosis for patients with foot drop. Int J Environ Res And Public Health. 2023;20(17):6687. 10.3390/ijerph20176687. 37681827 10.3390/ijerph20176687 PMC10487717

[43] Inc., T.M.: MATLAB version: 9.10.0 (R2021a). Natick, Massachusetts, United States: The MathWorks Inc; 2021. https://www.mathworks.com.

[44] Mallat R, Bonnet V, Dumas R, Adjel M, Venture G, Khalil M, Mohammed S. Sparse visual-inertial measurement units placement for gait kinematics assessment. IEEE Trans On Neural Syst And Rehabil Eng: A Publ Of The IEEE Eng In Med And Biol Soc. 2021;29:1300–11. 10.1109/TNSRE.2021.3089873. 10.1109/TNSRE.2021.3089873 34138711

[45] Pearl O, Shin S, Godura A, Bergbreiter S, Halilaj E. Fusion of video and inertial sensing data via dynamic optimization of a biomechanical model. J Biomech. 2023;155:111617. 10.1016/j.jbiomech.2023.111617. 37220709 10.1016/j.jbiomech.2023.111617

[46] Atrsaei A, Salarieh H, Alasty A. Human arm motion tracking by orientation-based fusion of inertial sensors and kinect using unscented kalman filter. J Biomechanical Eng. 2016;138(9):091005. 10.1115/1.4034170. 10.1115/1.4034170 27428461

[47] Camomilla V, Cereatti A, Vannozzi G, Cappozzo A. An optimized protocol for hip joint centre determination using the functional method. J Biomech. 2006;39(6):1096–106. 10.1016/j.jbiomech.2005.02.008. 16549099 10.1016/j.jbiomech.2005.02.008

[48] Gamage SSHU, Lasenby J. New least squares solutions for estimating the average centre of rotation and the axis of rotation. J Biomech. 2002;35(1):87–93. 10.1016/S0021-9290(01)00160-9. 11747887 10.1016/s0021-9290(01)00160-9

[49] Halvorsen K. Bias compensated least squares estimate of the center of rotation. J Biomech. 2003;36(7):999–1008. 10.1016/S0021-9290(03)00070-8. 12757809 10.1016/s0021-9290(03)00070-8

[50] Marin F, Mannel H, Claes L, Dürselen L. Accurate determination of a joint rotation center based on the minimal amplitude point method. Comput Aided Surg. 2003;8(1):30–34. 10.3109/10929080309146100 14708756 10.3109/10929080309146100

[51] Piazza SJ, Erdemir A, Okita N, Cavanagh PR. Assessment of the functional method of hip joint center location subject to reduced range of hip motion. J Biomech. 2004;37(3):349–56. 10.1016/S0021-9290(03)00288-4. 14757454 10.1016/s0021-9290(03)00288-4

[52] Siston RA, Delp SL. Evaluation of a new algorithm to determine the hip joint center. J Biomech. 2006;39(1):125–30. 10.1016/j.jbiomech.2004.10.032. 16271596 10.1016/j.jbiomech.2004.10.032

[53] Schwartz MH, Rozumalski A. A new method for estimating joint parameters from motion data. J Biomech. 2005;38(1):107–16. 10.1016/j.jbiomech.2004.03.009. 15519345 10.1016/j.jbiomech.2004.03.009

[54] Bräunig J, Wirth V, Kammel C, Schüßler C, Ullmann I, Stamminger M, Vossiek M. An ultra-efficient approach for high-resolution mimo radar imaging of human hand poses. IEEE Trans Intell Transp Syst On Radar Syst. 2023;1:468–80. 10.1109/TRS.2023.3309574.

