# Supplementary material for: IMU-based joint axis identification method for arbitrary joints in OpenSim - a simulation study
Source: BMC Biomed Eng. 2025 Nov 21;7:16. doi: 10.1186/s42490-025-00102-7 (PMC12639932; doi:10.1186/s42490-025-00102-7)
Supplement: Supplementary file 1 — Supplementary Material 1 [file 42490_2025_102_MOESM1_ESM.pdf]

Figure Captions

Result plots for noisy data filtered using a Butterworth filter

A1: ICOR coordinates over knee angle φ for noisefree and noisy data without outliers filtered using a Butterworth filter.

A2: Position of tibia in femur frame over knee angle based on noisy data, filtered using the Butterworth filter.
