# Supplementary figures and images for: IMU-based joint axis identification method for arbitrary joints in OpenSim - a simulation study
Source: BMC Biomed Eng. 2025 Nov 21;7:16. doi: 10.1186/s42490-025-00102-7 (PMC12639932; doi:10.1186/s42490-025-00102-7)

# ICOR coordinates over knee angle ( $\phi$ )

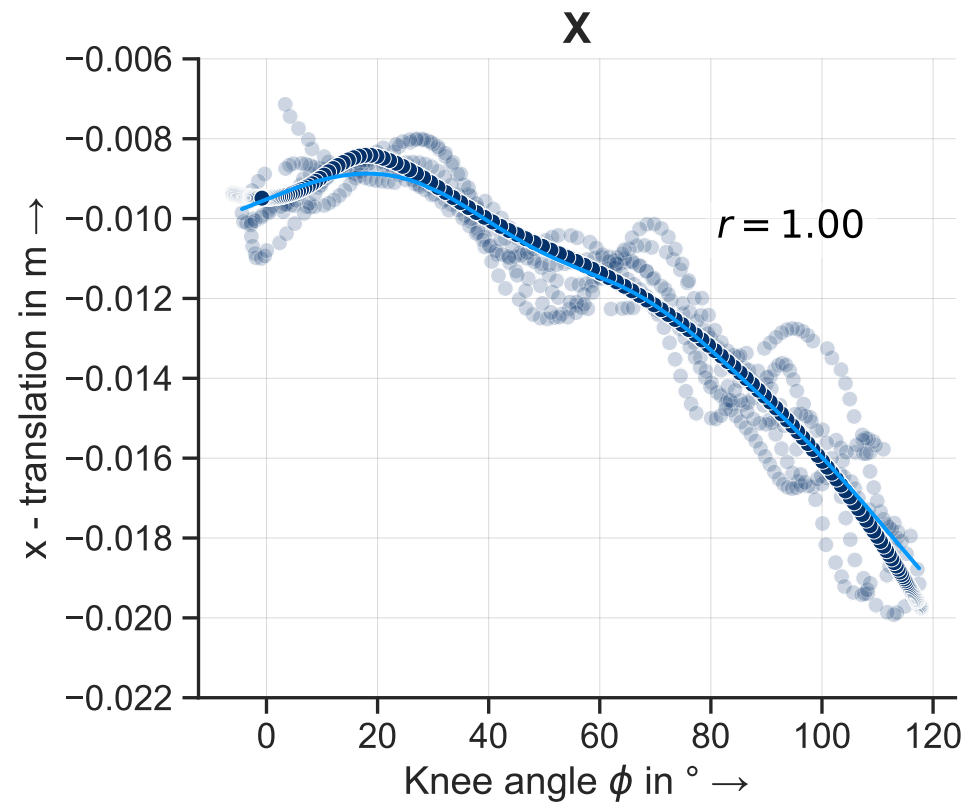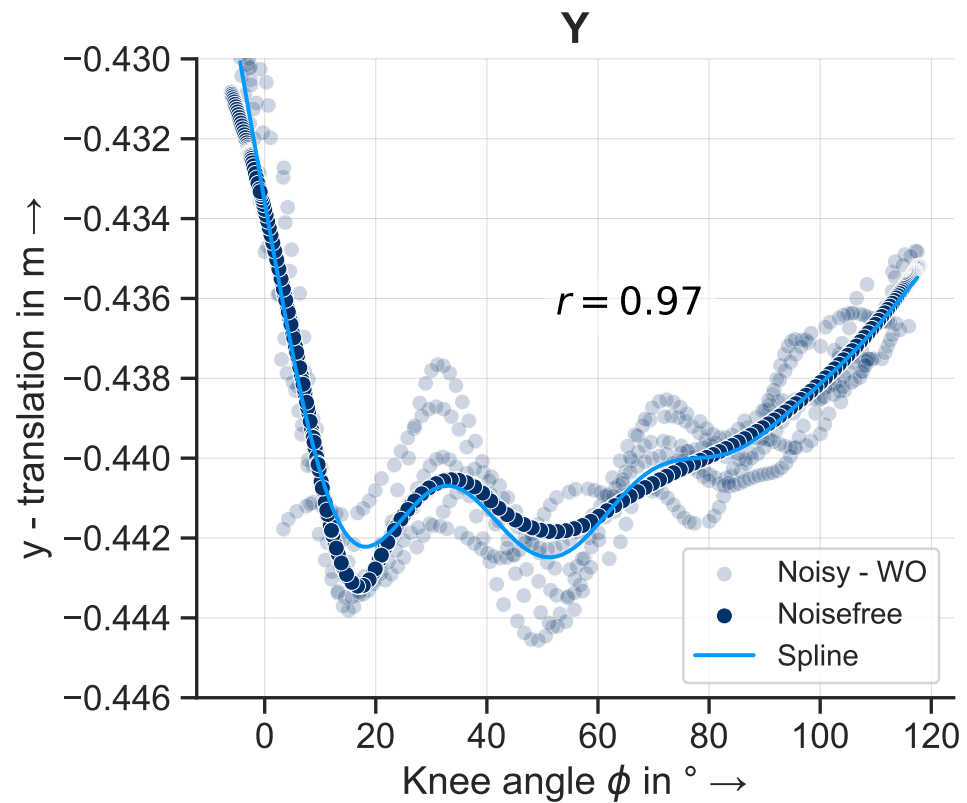

Supplement: Supplementary file 2 — Supplementary Material 2 [file 42490_2025_102_MOESM2_ESM.pdf]

# Position of tibia in femur - noisy data

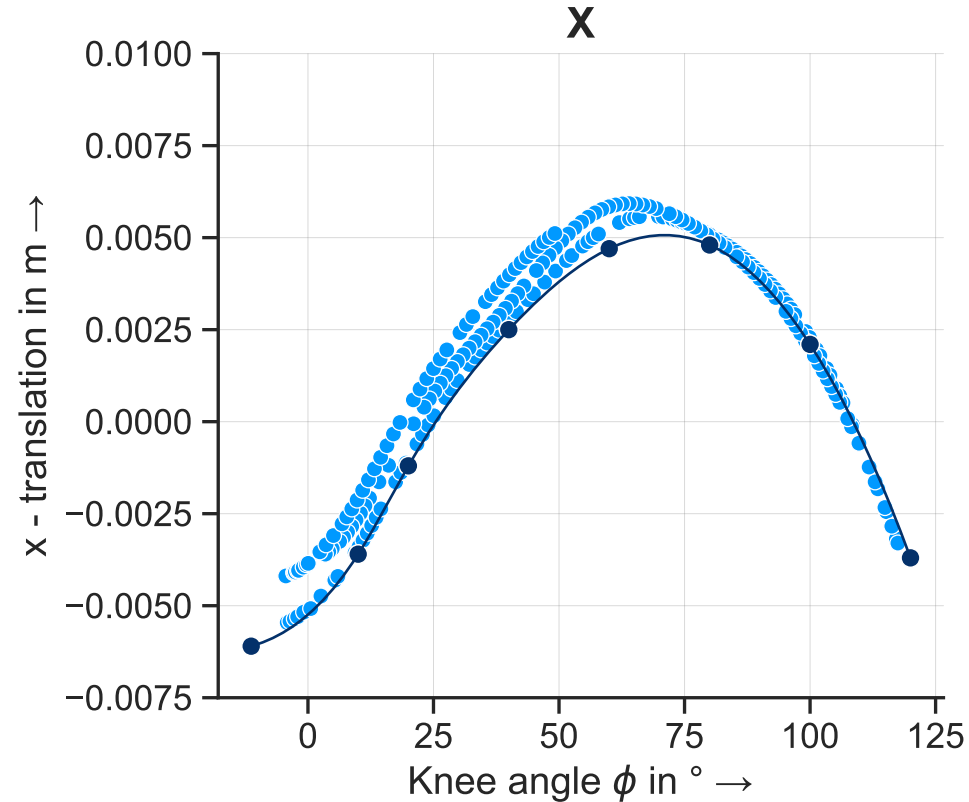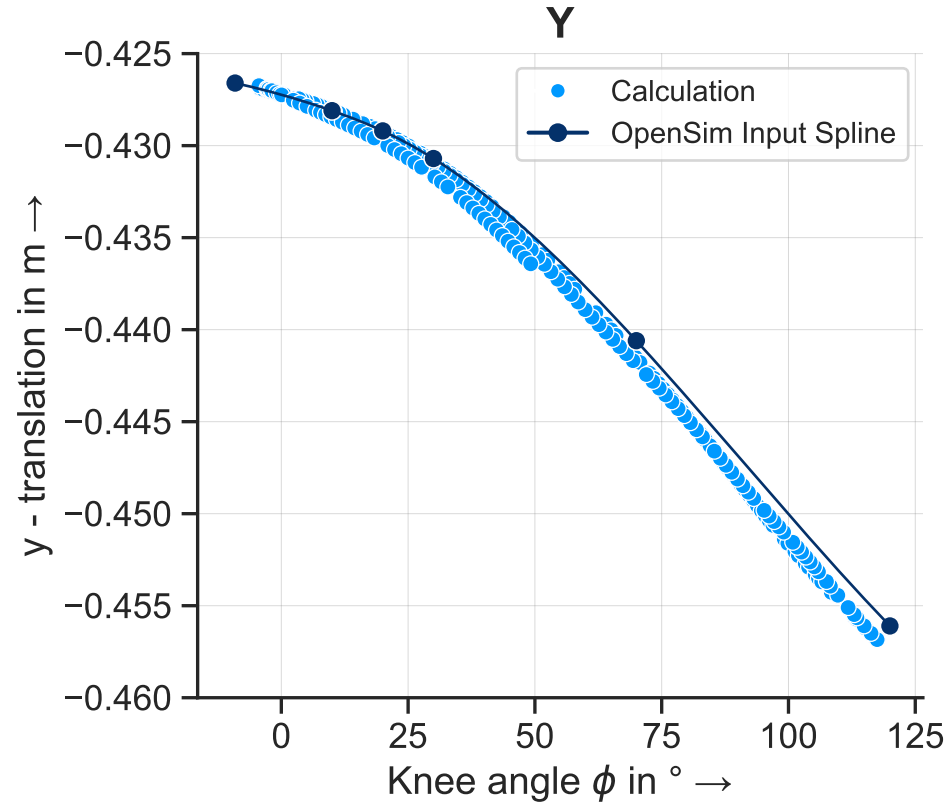

Supplement: Supplementary file 3 — Supplementary Material 3 [file 42490_2025_102_MOESM3_ESM.pdf]
